# Achieving Solar‐Thermal‐Electro Integration Evaporator Nine‐Grid Array with Asymmetric Strategy for Simultaneous Harvesting Clean Water and Electricity

**DOI:** 10.1002/advs.202303815

**Published:** 2023-09-22

**Authors:** Junli Ma, Zhenzhen Guo, Xu Han, Heng Lu, Kaixin Guo, Jianguo Xin, Chaoyong Deng, Xianbao Wang

**Affiliations:** ^1^ School of Integrated Circuits and Electronics Beijing Institute of Technology Beijing 100081 P. R. China; ^2^ School of Chemistry and Chemical Engineering Henan Institute of Science and Technology Xinxiang 473003 P. R. China; ^3^ School of Electronics & Information Engineering Guiyang University Guiyang 550005 P. R. China; ^4^ Hubei Collaborative Innovation Center for Advanced Organic Chemical Materials Ministry‐of‐Education Key Laboratory for the Green Preparation and Application of Functional Materials Hubei Key Laboratory of Polymer Materials School of Materials Science and Engineering Hubei University Wuhan 430062 P. R. China

**Keywords:** asymmetric strategy, nine‐grid evaporation/power generation array, solar‐driven interface water evaporation, solar‐thermal‐electro integration, water‐induced power generation

## Abstract

Water evaporation is a ubiquitous and spontaneous phase transition process. The utilization of solar‐driven interface water evaporation that simultaneously obtains clean water and power generation can effectively alleviate people's concerns about fresh water and energy shortages. However, it remains a great challenge to efficiently integrate the required functions into the same device to reduce the complexity of the system and alleviate its dependence on solar energy to achieve full‐time operation. In this work, a multifunctional device based on reduced graphene oxide (RGO)/Mn_3_O_4_/Al_2_O_3_ composite nanomaterials is realized by an asymmetric strategy for effective solar‐thermal‐electro integration that can induce power generation by water evaporation in the presence/absence of light. Under one sun irradiation, the solar‐driven evaporation rate and output voltage are 1.74 kg m^−2^ h^−1^ and 0.778 V, respectively. More strikingly, the nine‐grid evaporation/power generation array integrated with multiple devices in series has the advantages of small volume, large evaporation area, and high power generation, and can light up light‐emitting diodes (LEDs), providing the possibility for large‐scale production and application. Based on the high photothermal conversion efficiency and power production capacity of the RGO/Mn_3_O_4_/Al_2_O_3_ composite evaporation/generator, it will be a promising energy conversion device for future sustainable energy development and applications.

## Graphical abstract

A solar‐thermal‐electro integration evaporator is prepared with an asymmetric strategy and sandwich structure design. Solar water evaporation is combined with the electrokinetic effect to produce clean water and electric energy simultaneously. The integrated nine‐grid evaporation/power generation array achieves full‐time stable and efficient work without entirely relying on solar energy and has enhanced capacity and can easily power LEDs.

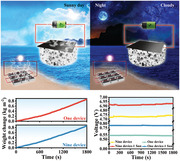

## 1 Introduction

Due to its abundant reserves, renewable and green, solar energy has broad application prospects in alleviating global problems such as the fossil energy crisis and the lack of freshwater resources.^[^ ^1^, ^2^, ^3^, ^4^ ^]^ Solar water evaporation technology based on interfacial heating can effectively concentrate thermal energy at the air‐water interface. By heating a thin layer of water at the water interface, clean water can be obtained through efficient photothermal conversion, realizing the utilization of renewable solar energy and abundant seawater. This technology has become a hot and widespread concern topic in the fields of seawater desalination and wastewater purification.^[^ ^5^, ^6^, ^7^, ^8^ ^]^ Nevertheless, the integrated desalination device still suffers from low energy conversion efficiency and unsatisfactory freshwater productivity in the initial research phase. To solve the above problems, photothermal utilization is a boost for ensuring adequate freshwater supply, and high energy conversion efficiency is achieved by suitable material selection and structural optimization. Moreover, clean water and electricity can be simultaneously generated to achieve a high energy conversion rate by combining solar thermal evaporation systems with energy collection technology.^[^ ^9^ ^]^ For example, Ho's team innovatively proposed the parallel collection of condensed water and frictional electricity during solar water evaporation. An Au nanoparticle hydrogel was prepared as a solar absorber, which showed an evaporation rate of 1.356 k m^−2^ h^−1^ under one sun irradiation. A small device was designed to generate frictional electrical energy by flowing condensed water on the PTFE surface, with a maximum voltage of ≈3 V.^[^ ^10^ ^]^ Zhou's group adopted a comprehensive energy utilization technology that uses solar energy for simultaneous desalination and extraction of electrical energy from evaporation‐induced salinity gradients and designed an integrated system based on carbon nanotubes (CNTs) modified filter paper and Nafion membrane. The system can achieve an evaporation rate of 1.15 kg m^−2^ h^−1^ and a voltage output of ≈0.062 V under 1 kW m^−2^ light intensity.^[^ ^11^ ^]^ Li’ group simultaneously obtained water evaporation and power generation by converting food waste (FW) into a highly porous carbon‐based photothermal material for low‐cost solar desalination, and FW combined with a thermoelectric module can achieve a stable water evaporation rate of 1.26 kg m^−2^ h^−1^ and a continuous voltage of 0.095 V under one sun.^[^ ^12^ ^]^

However, on the one hand, the problem of operation stagnation in the absence of solar illumination has not been fundamentally solved for most solar evaporation devices. On the other hand, most reported integrated systems tend to be assembled from independent work modules with complex and inflexible designs, which will harm the efficiency of solar evaporation and power generation. Recent theoretical and experimental studies have demonstrated that fluid flow in micro/nanochannels of surface‐charged materials (including CNTs, carbon black, graphene, metal oxide nanowires, layered double hydroxides, and biological fibers) may induce unique electrokinetic charge transfer for power generation by forming a double charge layer (EDL) at the interface between the fluid and the channel wall.^[^ ^13^ ^]^ Nevertheless, a prerequisite for achieving power generation is the existence of an ion gradient in the device. Unfortunately, the fluid in a solar interface evaporation system is uniformly distributed and transmitted on the surface of the light‐absorbing layer.^[^ ^14^ ^]^ Therefore, the distribution of photothermal materials in the evaporation system should be regulated to promote the formation of ion gradient distribution/transport in the nanochannels, which is a necessary means to achieve continuous and efficient power generation. Simultaneous water evaporation and power generation can be carried out in a good integrated way without reducing the efficiency of solar evaporation conversion.

**Figure 1:**
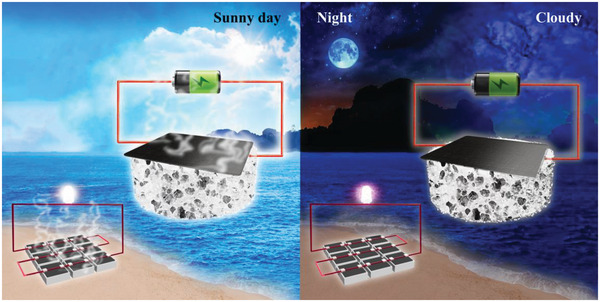
Schematic of A‐RMA/GO@P‐SA evaporator/generator full‐time operation under sunlight and environmental conditions.

In this study, we report a multifunctional device based on reduced graphene oxide (RGO)/Mn_3_O_4_/Al_2_O_3_ (RMA) composite nanomaterials for effective solar‐thermal‐electro integration through an asymmetric strategy. RGO has been successfully applied as a highly efficient solar photothermal material in solar thermal conversion due to its inherent light stability and low toxicity. However, RGO has poor compatibility as a light‐absorbing material.^[^ ^15^, ^16^ ^]^ Mn_3_O_4_ has attracted much attention because of its low cost, environmental friendliness, high stability, and theoretical specific capacitance, but poor conductivity limits its application.^[^ ^17^, ^18^ ^]^ The composite material RMA composed of RGO, Mn_3_O_4_, and Al_2_O_3_ with high thermal conductivity can form a synergistic effect that can improve the conductivity, structural stability, and thermal conductivity of the composite material.^[^ ^19^, ^20^ ^]^ It provides the possibility for efficient simultaneous solar water evaporation and power generation. Therefore, solar evaporators composed of RMA composites uniformly loaded on air‐laid paper and sodium alginate (SA) aerogels can achieve an efficient evaporation rate of 1.88 kg m^−2^ h^−1^, the solar photothermal conversion efficiency can reach 94.3%, and the devices are easy to prepare (room temperature preparation), environmentally friendly, and low‐cost. Based on the excellent photothermal performance of RMA composites, a sandwich film strategy was adopted to load RMA and GO in a nonuniform layering on air‐laid paper to form an RMA/GO/RMA layout, which expanded the asymmetry of the film in different aspects. As a result, the asymmetric RMA/GO@air‐laid paper (A‐RMA/GO@P‐SA) evaporator/generator can achieve efficient power generation under ambient conditions and solar illumination, and has the ability to work full‐time without entirely relying on solar energy. Note that the power generation capacity shows a remarkable enhancement and the synchronous solar evaporation rate remains very efficient under solar irradiation. In addition, the assembly of integrated equipment is realized for the first time. The nine‐grid evaporation/power generation array constructed by nine devices in series has the characteristics of small space occupation, flexibility, and portability, and can realize the work capacity under light conditions both in experimental conditions and outdoors. Furthermore, this integration mode not only increases the evaporation area, but also improves the power production capacity (**Figure** **1**). The nine‐grid evaporation/power generation can output voltages up to 6.917 V and successfully light an LED, which offers the possibility of large‐scale seawater desalination and energy power generation.

## 2 Results and Discussion

**Figure 2:**
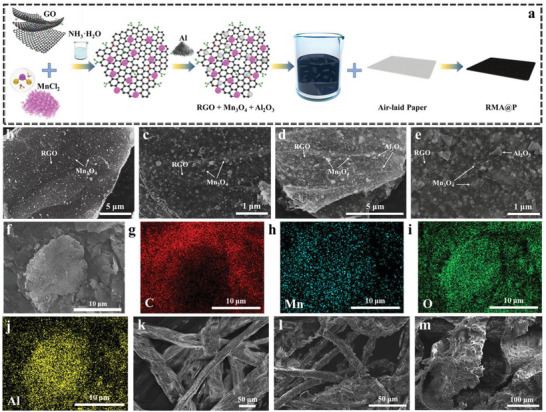
a) The synthetic process of RMA at room temperature. b,c) SEM images of RM. d,e) SEM images of RMA. f–j) SEM image and corresponding elemental mapping images of C, Mn, O, and Al elements of RMA. k,l) SEM images of the blank film and the RMA@P film. m) SEM images of SA.

The process of spontaneous reduction and assembly of RGO/Mn_3_O_4_‐based composite films at room temperature is shown in (**Figure** **2**a). MnCl_2 _· 4H_2_O was added to the GO suspension containing NH_3_· H_2_O, and Mn_3_O_4_ nanoparticles grew on the GO sheet layer. RGO/Mn_3_O_4_/Al_2_O_3_ composites were prepared by adding aluminum powder to the above solution. The microstructure and morphology of the RGO/Mn_3_O_4_‐based composites were characterized by scanning electron microscopy (SEM) (Figure 2b,d). Due to the strong interaction between the surface functional groups of GO and Mn_3_O_4_ nanoparticles, Mn_3_O_4_ nanoparticles with block structure could be tightly adsorbed to GO.^[^ ^21^ ^]^ In Figure 2c,e, the ripples and wrinkles on the surface of RGO can be observed, demonstrating that GO was reduced to RGO, which can provide more active sites. Therefore, Mn_3_O_4_ nanoparticles were induced to adhere to the surface of RGO sheets.^[^ ^18^ ^]^ Moreover, Al_2_O_3_ nanoparticles were also distributed on RGO in the RMA composites. The consistent characteristics of the nanocomposites are attributed to the fact that graphene sheets can effectively inhibit the aggregation of metal oxides, resulting in better dispersion of Mn_3_O_4_ nanoparticles on the surfaces.^[^ ^22^ ^]^ In addition, through the surface analysis of the elements in the selected area, such as Figure 2f–j, C, Mn, O, and Al elements are distributed on the surface of RMA, and the surface analysis of RM elements is shown in Figure S1 (Supporting Information). Figure S2 (Supporting Information) shows the X‐ray energy dispersive spectroscopy (EDS) spectrum, which further indicates the elemental composition of the synthesized composite. To investigate the structure and morphology of the samples, transmission electron microscopy (TEM) images of RM and RMA were studied. Figure S3a,b (Supporting Information) shows the close contact between the RGO sheet and Mn_3_O_4_ nanoparticles, the uniform distribution of Mn_3_O_4_ on the RGO sheet, and the size of Mn_3_O_4_ nanoparticles in the range of 10–50 nm. Meanwhile, the presence of Al_2_O_3_ is demonstrated.

Subsequently, the air‐laid paper was immersed in a uniformly dispersed RMA suspension, and the uniform state of the RMA suspension facilitated the acquisition of a uniform RMA composite film during the assembly process. Figure S4a (Supporting Information) shows an optical picture of the blank air‐laid paper. The blank film is white. Then, we tested the SEM of the blank film and the RMA film, and the results are shown in Figure 2k,l. The blank film presents a paper‐fibrous structure with a smooth surface. After loading the RMA composite material, the white blank film transformed to black, as shown in Figure S4b (Supporting Information). From the SEM image (Figure 2l), the surface of the RMA film is rougher, the paper fiber nanowires are covered with RMA composites, and the RMA nanosheets are tightly attached to the surface of the nanowires.

To ensure thermal management and water transportation in the solar photothermal conversion system, 3D aerogels were prepared with SA and calcium carbonate as raw materials to serve as the thermal insulation, support layer, and water channel of the solar thermal conversion system in this work. SA that is environmentally friendly is an edible binder and can easily form ionic cross‐links with Ca^2+^ to obtain water‐insoluble porous gel‐like structures.^[^ ^23^ ^]^ Figure S5 (Supporting Information) shows the synthesis process of the SA porous system. SA and CaCO_3_ were uniformly mixed in deionized water, and CaCO_3_ provided a source of Ca^2+^ ions. Subsequently, gluconate lactone was added to the mixed solution, which reacted with CaCO_3_ to release Ca^2+^. After vertical freezing and freeze drying, the 3D aerogels with water‐insoluble porous gel‐like structures were obtained (Figure S6, Supporting Information). SEM shows that the 3D aerogel has abundant pores and cells, and the size is between micron and submicron. The 3D porous structure can ensure that water can be continuously delivered to the evaporator, and promote salt ion exchange, thereby preventing the accumulation of salt in seawater in the support layer (Figure 2m; Figure S7, Supporting Information). When the evaporator was placed on the water surface, the water was immediately transported to the top of the evaporator through the interconnected and continuous pores in the evaporator. The water absorption properties of SA aerogels were studied. The results showed that the aerogels can absorb 8.25 g of water in 1 min (Figure S8, Supporting Information). The strong wicking effect ensures that SA‐based 3D aerogel possesses excellent water transport capability. In Figure S9 (Supporting Information), the MO placed on the surface of the evaporator quickly dissolved and entered the bottom liquid. After 2 min, the water below started turning yellow, and the color gradually deepened with time, fully demonstrating the outstanding water supply and ion exchange capabilities of the aerogel. For the thermal insulation water supply layer, in addition to ensuring sufficient water supply, the heat loss should be reduced as much as possible. The thermal conductivity of RMA is 0.025 W m^−1^ K^−1^, which is significantly lower than that of water (0.59 W m^−1^ K^−1^).^[^ ^24^ ^]^ Therefore, the light absorption layer can maintain continuous and stable local heating during the evaporation process, thereby minimizing heat dissipation and achieving a high evaporation rate. Meanwhile, 3D aerogels have very low density (0.06 g cm^−3^), indicating that the aerogels have great application potential in the fields of light weight. The 3D aerogel can realize continuous water supply and effective salt ion exchange. Therefore, SA 3D aerogels with many excellent properties can be used as an ideal heat insulation layer and water channel in solar photothermal evaporation devices.

**Figure 3:**
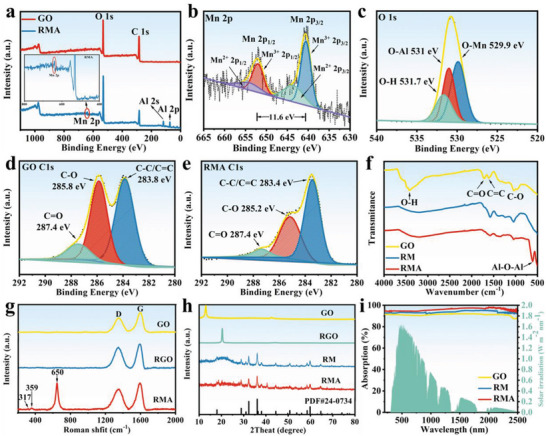
a) XPS spectra of GO and RMA, the illustration is the local amplification of Mn 2p peak in the RMA XPS spectrum. b) XPS spectra of Mn 2p. c) XPS spectra of O 1s. d) XPS C1s spectra of GO. e) XPS C1s spectra of RMA. f) FT–IR spectra of GO, RM, and RMA. g) Raman spectra of GO, RGO, and RMA. h) XRD patterns of GO, RGO, RM, and RMA. i) Light absorption capabilities of GO, RM, and RMA.

To determine the chemical composition and chemical bond state of RMA composites, the samples were investigated by X‐ray photoelectron spectroscopy (XPS). The complete detection spectrum of the compound in **Figure** **3**a confirmed that Mn, O, C, and Al exist in the RMA composites without other impurities. In the spectrum of the RMA composite, the corresponding Mn 2p, O 1s, C 1s, Al 2p, and Al 2s peaks are ≈640.4, 531.8, 284.5, 73.7, and 119.5 eV, respectively.^[^ ^25^, ^26^, ^27^ ^]^ The XPS spectra of Mn 2p in RMA are shown in Figure 3b. The two peaks located at 640.6 and 652.2 eV are consistent with the Mn 2p_3/2_ and Mn 2p_1/2_ spin‐orbit peaks of Mn_3_O_4_,^[^ ^17^ ^]^ and the energy gap between the two energy levels is 11.6 eV, which is consistent with previous studies reported on Mn_3_O_4_,^[^ ^28^ ^]^ indicating the successful preparation of Mn_3_O_4_.^[^ ^29^ ^]^ Meanwhile, the Mn 2p peaks were deconvoluted into four peaks located at 640.6, 652.1, 643.9, and 655.1 eV, which are attributed to Mn^3+^ and Mn^2+^, respectively. Note that the corresponding peak area ratio of Mn^3+^ and Mn^2+^ was close to 1:2, which confirmed the formation of Mn_3_O_4_.^[^ ^30^ ^]^ Figure 3c shows the XPS spectrum of O 1s, which can be decomposed into three components, namely the O─Mn bond at 529.9 eV, the O─Al bond at 531 eV, and the O─H bond at 531.7 eV,^[^ ^25^ ^]^ combined with the Al 2p peak located at 73.7 eV and the Al 2s peak at 119.5 eV, indicating the presence of Al_2_O_3_ in the RMA composite. The conversion of GO to RGO can be observed by comparing the binding energy spectrum of RMA with the C1s of GO. In the C1s spectrum of GO, three peaks are located at 283.8, 285.8, and 287.4 eV, representing the peaks of C─C/C═C, C─O, and C═O, respectively (Figure 3d).^[^ ^26^ ^]^ The peak intensities of C─O and C═O in the C1s spectrum of the RMA composite both show a significant decrease, indicating that some of the oxygen‐containing groups on the surface of GO were removed and GO was successfully reduced to RGO (Figure 3e).^[^ ^24^ ^]^ To analyze the chemical structure of GO, RM, and RMA composites, FT–IR patterns are shown in Figure 3f. The FT–IR spectra of GO reveal the presence of O─H (3411 cm^−1^), C═O (1718 cm^−1^), C─C/C═C (1612 cm^−1^), and C─O (1035 cm^−1^).^[^ ^31^, ^32^ ^]^ In the FT‐IR spectra of RM and RMA, the stretching vibration peaks of hydroxyl groups on the surface of GO were removed. In contrast, RM and RMA also have reduced C═O content, which means a reduction in GO.^[^ ^20^ ^]^ The characteristic peaks of oxygen‐containing functional groups of GO were significantly weakened or completely eliminated, indicating that GO was successfully reduced to RGO during the reaction. The peak at 606 cm^−1^ was caused by the vibration of the Al─O─Al bond, which further proved the successful formation of Al_2_O_3_ in RMA.^[^ ^33^ ^]^

The properties of carbon‐containing materials can be effectively characterized by Raman spectroscopy for the verification of GO to RGO conversion.^[^ ^34^ ^]^ The Raman spectra of GO, RGO, and RMA are shown in Figure 3g. Among them, the bands located at 1342 and 1597 cm^−1^ of the GO Raman spectra correspond to the vibration modes of the dangling bonded carbon atoms (*A*_1g_ vibration) in the disordered band (D band) and the vibration modes of all sp^2^‐bonded carbon atoms (*E*_2g_ vibration) in the ordered graphite band (G band), respectively. The intensity ratio between the D peak and G peak is related to the nature of the carbon.^[^ ^35^, ^36^, ^37^ ^]^ The *I*_D_/*I*_G_ values of RGO and RMA composites are 0.87 and 0.77, respectively, which are higher than the value of 0.72 for GO. This indicates that a reduction reaction occurred during the assembly process and a higher degree of graphitization of RGO, which is beneficial to improving the electrical conductivity of RMA nanocomposites.^[^ ^18^ ^]^ Note that the main scattering peak at 650 cm^−1^ and the secondary scattering peaks at 317 and 359 cm^−1^ in RMA are characteristic of the Mn─O bond vibration mode, revealing the successful synthesis of Mn_3_O_4_ in the composites.^[^ ^38^ ^]^ To study the crystal structures of the different materials, measurements were performed by X‐ray diffractometry, as shown in Figure 3h. In RM and RMA, the XRD patterns of Mn_3_O_4_ in the synthesized state show strong diffraction peaks, indicating that it has a high degree of crystallinity. The diffraction peaks indicate that Mn_3_O_4_ has a tetragonal structure (JCPDS, No. 24–0734), the lattice constants are a = b = 5.76 Å, c = 9.47 Å, and the space group is *I*41/*amd*.^[^ ^39^ ^]^ The positions of the main diffraction peaks at ≈18.11°, 28.92°, 31.02°, 32.32°, 36.12°, 38.02°, 44.47°, 50.77°, 59.91°, and 64.5° correspond well to the (101), (112), (200), (103), (211), (004), (200), (105), (224), and (400) crystal planes of the Mn_3_O_4_ phases, respectively. The characteristic peaks of Al_2_O_3_ are also detected. Furthermore, by comparing the XRD patterns of GO and RGO, the peak appearing near 2*θ*≈20.41° matches the characteristic peak (002) of graphene. Thus, a large number of oxygen‐containing functional groups in GO have been removed during the chemical reduction process, implying the reduction of GO to RGO.^[^ ^40^ ^]^ Therefore, it can be clearly illustrated that the RMA composites are composed of RGO, Mn_3_O_4_, and Al_2_O_3_.

Effective light harvesting performance is a prerequisite for achieving efficient solar evaporation. The light absorbance of different samples was tested by UV‒vis‒NIR spectrophotometry, as shown in Figure 3i. RMA showed a light absorption of more than 90% in the wavelength range from 200 to 2500 nm. The high optical absorption performance of RMA was primarily due to the synergistic effect between the composite materials. Compared to the Raman spectra and FTIR spectra of RM (Figure 3f; Figure S10, Supporting Information), RMA exhibited a higher degree of RGO reduction. Additionally, the SEM images of RM and RMA are shown in Figure S11 (Supporting Information). The surface of RM has a smoother appearance with a lower degree of RGO reduction, while the surface of RMA exhibits a very obvious hierarchical layered structure. This structure enables internal refraction and reflection of light, increasing the propagation path of light and reducing the reflection of light. As a result, RMA demonstrates higher optical absorption performance. With such a good light absorption capacity, RMA is expected to achieve an efficient solar photothermal conversion.

**Figure 4:**
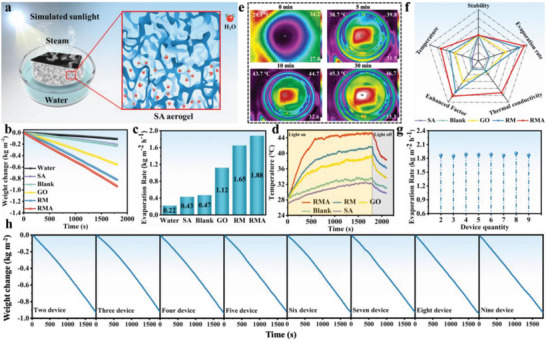
a) Schematic diagram of photothermal evaporation performance test. b) Weight change of the photothermal evaporation system for different devices. c) Evaporation rate of the photothermal evaporation system for different devices. d) Surface temperature of different evaporation devices. e) Thermal image of RMA@P‐SA. f) Comparisons of solar‐driven interfacial evaporation performance. g) Evaporation rate of different quantities of devices. h) Weight change of the photothermal evaporation system for the quantity of device.

A schematic diagram of the RMA@P‐SA solar evaporation system is shown in **Figure** **4**a. The photothermal evaporation performance was tested under one sun through a real‐time photothermal evaporation measurement system. In the combination of RMA@P and the 3D aerogel, the photothermal material RMA could effectively absorb the incident solar energy and convert it into thermal energy, creating a localized heating zone. Because of its rich pore structure, the adjacent pores of the 3D aerogel are connected to form well‐interpenetrated low‐curvature pore channels. It can be regarded as a microwater reservoir, which is more conducive to water transport and salt ion exchange. Additionally, 3D aerogels have extremely low thermal conductivity and low density, which can not only effectively suppress the heat loss from interfacial thermal conduction to the bulk water below, but also ensure that the entire system floats on the water, so that the evaporation process can be carried out continuously and stably. To investigate the water evaporation capacity of solar evaporators with different structures, the evaporation curves of RMA@P‐SA, RM@P‐SA, GO@P‐SA, blank air‐laid paper‐SA, SA, and pure water with time under one solar irradiation were plotted, as shown in Figure 4b. RMA@P‐SA evaporated the most water per unit area compared to the other evaporators. The calculated evaporation rates of different photothermal devices are shown in Figure 4c. As a blank control, the evaporation rates of SA and air‐laid paper solar evaporation systems without photothermal materials were 0.43 and 0.47 kg m^−2^ h^−1^ under 1 sun, respectively. Meanwhile, the pure water evaporation rate was also investigated to exclude its effect. The results show that the RMA‐based solar evaporator had an excellent water evaporation rate of 1.88 kg m^−2^ h^−1^, which was superior to the other two evaporators and was nine times higher than the pure water evaporation rate (0.22 kg m^−2^ h^−1^) (Figure S12, Supporting Information).

The corresponding solar‐to‐thermal conversion efficiency (*η*) can be estimated by the following equation

(1) $$\eta \;\;=\;\;\frac{mH_{\mathrm{LV}}}{q_{\mathrm{solar}}}$$

where η is the solar‐to‐vapor photothermal conversion efficiency, *m* is the net evaporation rate after subtracting the evaporation rate in the dark from the evaporation rate under light conditions, and *H*_LV_ is the total enthalpy change from water to vapor (including latent heat and sensible heat). The standard dimension is kJ kg^−1^, which is determined by Calculation S1 (Supporting Information), and *q*_solar_is the simulated solar energy intensity (1 kW m^−2^). Therefore, the calculated solar photothermal conversion efficiency was up to 94.3% under one sun. Since there is an unavoidable heat loss process during evaporation, the radiation loss, transmission loss, and convection loss of the RMA@P‐SA evaporation system were calculated as shown in Calculation S2 (Supporting Information). Temperature changes were recorded to test the photothermal behavior of solar evaporators composed of different materials under one sun irradiation. As shown in Figure 4d, the surface temperature of the evaporator rapidly increased in the first 500 s. As the evaporation time is prolonged, the temperature gradually tends to be stable without significant fluctuations. The surface temperature of RMA@P remained constant at 45.3 °C, which was higher than the steady–state temperature of other solar absorbers under the same irradiation time. This indicates that RMA@P exhibits a rapid thermal response. However, the surface temperature of the solar evaporator gradually decreased as the light source was turned off. In addition, the surface temperature of the RMA@P‐SA photothermal device increased rapidly and reached a steady‐state temperature of 45.3 °C after 30 min of illumination (Figure 4e), showing the rapid photothermal response. The above results show that the RMA@P‐SA evaporator achieved excellent photothermal conversion, timely water supply, and effective thermal management. The improvement in evaporation performance was attributed to the synergistic effect generated by the introduction of Al_2_O_3_ and the excellent thermal management performance of SA. The formation of Al_2_O_3_ significantly strengthened the thermal conductivity of RMA composites. The thermal conductivity of RM is 0.139 W m^−1^ K^−1^, while the thermal conductivity of RMA (1.425 W m^−1^ K^−1^) is ten times higher than that of RM (Figure S13, Supporting Information). The high thermal conductivity of the RMA composites could enhance the heat transfer from the RMA@P surface to the interface water, which promoted water evaporation to improve the photothermal conversion efficiency. Moreover, the SA aerogel with low thermal conductivity could reduce the heat loss caused by the heat transfer from the interface heating zone to the bulk water below.

In general, the RMA@P‐SA evaporator achieves significant and comprehensive advantages over other evaporation systems (Figure 4f). Figure 4g,h shows the evaporation rate and mass change of water for 2–9 RMA@P‐SA evaporators in the 1800s under one sun irradiation, respectively. The evaporators were tested for 20 cycles and always maintained a stable evaporation rate (Figure S14, Supporting Information), which fully proves the photothermal stability, reusability, and durability of the RMA@P‐SA evaporators. In addition, the salt tolerance of the RMA‐P@SA evaporator was also evaluated. The evaporator was placed in high‐concentration brine (10 wt.%) and subjected to continuous photothermal evaporation testing for 24 h. As shown in Figures S15 and S16 (Supporting Information), the evaporation rate of the system remained stable during the evaporation process in high‐concentration brine, with an average evaporation rate of 1.8 kg m^−2^ h^−1^. There were no salt crystallizations observed on the surface of the evaporator, ensuring that the evaporation rate was not affected (Figure S17, Supporting Information).

**Figure 5:**
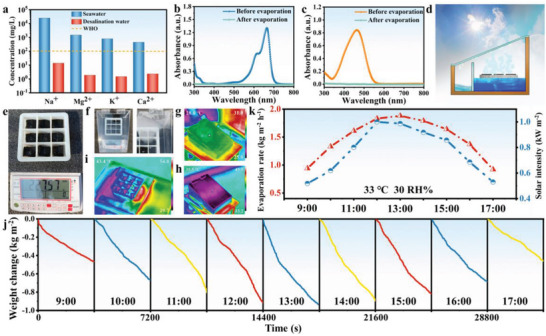
a) Main ion (Na^+^, Mg^2+^, K^+^, and Ca^2+^) concentrations of seawater before and after desalination. b) Methylene blue and c) methylene orange solutions before and after purification. d) Schematic diagrams of a solar‐driven interfacial evaporator prototype. e,f) Photographs of solar steam generation and collection using the RMA membrane array under outdoor conditions. g–i) Thermal images of the solar‐driven interfacial evaporator before and after sunlight illumination. j) Weight change of the photothermal evaporation system during different time periods under natural sunlight. k) The solar intensity spectra, ambient air temperatures, ambient relative humidity, and synchronous evaporation rates of the RMA membrane array‐based solar still from 8:00 a.m. to 5:00 p.m. on 17 September 2022 at Beijing Institute of Technology.

As a green process, the operability of solar photothermal evaporation technology in seawater desalination and wastewater treatment is of great importance for practical applications. The concentrations of sodium (Na^+^), magnesium (Mg^2+^), potassium (K^+^), and calcium (Ca^2+^) in real seawater and desalinated samples were measured by inductively coupled plasma‒optical emission spectrometry (ICP‒OES). As shown in **Figure** **5**a, the ion concentrations (Na^+^, Mg^2+^, K^+^, and Ca^2+^) in the water during desalination significantly decreased from 24 970, 1491, 801, and 450.3 ppm to 13.79, 1.892, 1.486, and 2.334 ppm, respectively. The ion concentrations in the desalted water were much lower than the drinking water standards set by the World Health Organization (WHO), indicating that the photothermal evaporation of RMA@P‐SA could effectively remove four ions from the original solution. The practical application of freshwater production can be realized through the ion rejection of the RMA@P‐SA evaporator. Figure 5b,c shows the absorption spectra of methylene blue and methyl orange solutions before and after purification. The water collected after evaporation did not show the characteristic absorption peaks of methylene blue and methyl orange, which confirmed that solar evaporation could effectively remove the dyes from the wastewater. All these results demonstrate that the RMA@P‐SA evaporator can be applied to seawater desalination and wastewater treatment.

To verify the practical feasibility of the hybrid solar evaporation system, the hybrid system was placed in a natural light environment to test its continuous evaporation performance. We designed a portable interfacial solar evaporator prototype, as shown in Figure 5d, which can be applied to collect pure water on land. The RMA@P‐SA‐based nine‐grid portable prototype was placed in an outdoor environment, and the mass changes during evaporation were recorded (Figure 5e,f). Real‐time monitoring of outdoor solar intensity was carried out, and the corresponding ambient temperature, relative humidity, and evaporation rate as well as the evaporation curve over time were recorded at the same time (Figure 5j,k). The evaporation performance of the solar evaporator was enhanced with increasing light intensity. The evaporation rate of the RMA@P‐SA‐based evaporation system showed a peak with a maximum rate of 1.85 kg m^−2^ h^−1^ from 12:00 a.m. to 2:00 p.m. During the cloudy period, the evaporation rate decreased slightly due to the weak sunlight intensity. With the recovery of sunlight intensity, the evaporation rate increased rapidly because of the rapid interface positioning heating of RMA@P. Under sunlight irradiation, a large amount of water evaporated rapidly, and subsequently, the vapor condensed on the surface of the condenser wall to collect clean water (Figure 5f). The infrared images show the apparent thermal localization of RMA@P to efficiently generate steam (Figure 5g–i). In practical applications of the solar‐driven interfacial evaporator, the ultimate performance of the device depends on the solar intensity and other environmental factors. From 8:00 a.m. to 5:00 p.m. on 17 September 2022, a full‐day outdoor test was conducted in Beijing to evaluate the photothermal evaporative performance of the solar thermal evaporation system based on the RMA membrane array. The net water collection rate (total water yield) of the evaporation system was ≈7.39 kg m^−2^. The results indicate that the device can provide enough daily water for two adults using only 1 square meter of the system in one day. This demonstrates the effective condensation of the thermal vapor and highlights the extensive commercial potential of the device.

**Figure 6:**
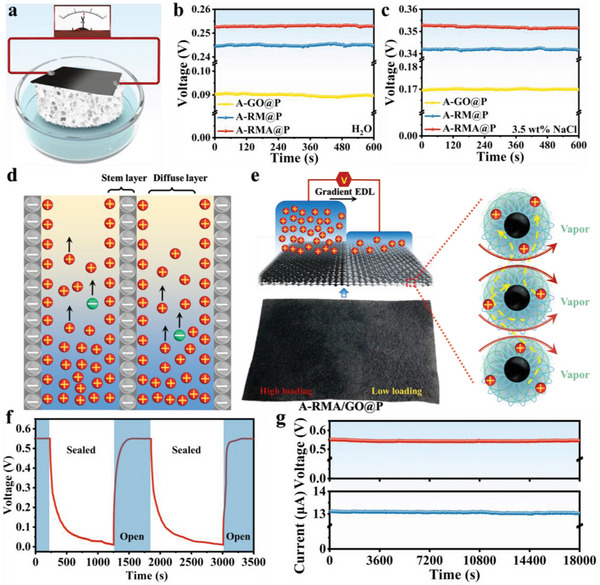
a) Schematic diagram of water‐induced energy generation of different devices under ambient conditions. b,c) Voltage output from the A‐GO@P, A‐RM@P, and A‐RMA@P generators immersed in water and 3.5 wt.% NaCl under ambient conditions. d) Schematic of ion transport within the EDL channels. e) Schematic illustration of the voltage generation from the asymmetrical load device. f) The measured voltage output of the device when the beaker was periodically sealed and open. g) Long‐term output measurement over 6 h with the A‐RMA/GO@P device under ambient conditions.

With the study of solar water evaporation, people have proposed combining the solar water evaporation process with power generation to simultaneously solve the problem of clean water and energy shortages.^[^ ^41^ ^]^ Therefore, the water evaporation‐induced power generation performance of the RMA@P‐based solar evaporator was investigated. GO@P, RM@P, and RMA@P were placed in deionized water and used to test the water‐induced power generation performance, as shown in **Figure** **6**a. In Figure S18 (Supporting Information), the GO, RM, and RMA devices uniformly loaded on air‐laid paper generated only 0.05, 0.13, and 0.135 V, respectively, at room temperature. The stable open‐circuit voltages of the asymmetrically loaded GO@P (A‐GO@P), RM@P (A‐RM@P), and RMA@P (A‐RMA@P) devices were 0.09, 0.245, and 0.253 V, respectively (Figure 6b). Then, they were placed in NaCl solution (3.5 wt.%). The improvement in the output voltage of the three generators can be observed in Figure 6c, where A‐RMA@P produced the largest voltage, reaching up to 0.352 V. According to previous experiments and reports, water‐induced power generation is based on the electrokinetic effect.^[^ ^42^ ^]^ As shown in Figure S19 (Supporting Information), the zeta potential measurements of GO, RM, and RMA are all negative, indicating that the surfaces of the materials are negatively charged. The negatively charged surfaces will repel ions with the same charge polarity (OH^−^ or Cl^−^) and attract ions of opposite polarity (H_3_O^+^ or Na^+^) at the same time, implying that the main charge carriers are H_3_O^+^ and Na^+^, respectively, in deionized water and NaCl solutions. According to the EDL theory, a thin layer of counter ions (H_3_O^+^ or Na^+^) will be attached to the negatively charged channel wall to form a Stern layer, and the ions away from it will collectively form a Diffusion layer,^[^ ^43^ ^]^ that is, an overlapping EDL is formed on both sides of the nanochannel, as shown in Figure 6d. When water flows through a negatively charged channel, anions with the same charge polarity (OH^−^ or Cl^−^) will be repelled, while more polar opposite ions (H_3_O^+^ or Na^+^) will be attracted, and preferentially pass the channel, resulting in a higher potential downstream. Continuous evaporation can drive consecutive capillary flow in the channel, creating a successive potential difference based on the streaming potential effect. The level of zeta potential directly determines the interaction between the material the liquid and the power output. The high zeta potential can attract more charges into the EDL.^[^ ^14^ ^]^ Thus, A‐RMA@P has a stronger power generation capacity. For materials with uniformly distributed surfaces, it is almost impossible to form ion concentration gradients, resulting in a smaller potential difference and a lower power generation capacity. Therefore, an asymmetric charge distribution was achieved by a nonuniformly loaded evaporator to improve the power generation capacity, as shown in Figure 6e. Due to the inhomogeneous load, the charges formed an asymmetric distribution in the film, which led to different ion adsorption behaviors in the EDL at different positions of the film. The side of the high‐quality loaded RMA acted as an electron collector, and the strong ionic attraction made cations gather on this side and repel the anions. To maintain ion balance, the anions were distributed in the remaining regions with low loading mass. Thus, the load gradient in the film caused significant differences in ion adsorption capacity. The dominant charge carriers (H_3_O^+^ or Na^+^) moved upward through the negatively charged RMA channel walls and formed a unique gradient EDL and potential difference via the nanochannel network to induce the directional movement of charges, generating a flow current for the external output of electric energy.

Therefore, we tried to increase the load gradient of the film to expand the asymmetry of the film. A GO layer was nonuniformly loaded on the A‐RMA@P film to form an asymmetric film with an RMA/GO/RMA sandwich structure (A‐RMA/GO@P). The introduction of the GO layer led to differences in the oxygen‐containing functional groups between the different film layers, so there was also a certain concentration gradient of the functional groups between the film layers.^[^ ^44^, ^45^ ^]^ This further strengthened the asymmetry of the film to obtain an expanded distribution gradient EDL and potential difference to improve the power generation capability of the device. As shown in Figure S20 (Supporting Information), the zeta potential of the mixture of RMA and GO (mass ratio 2:1) reached −52.9 mV, indicating that the addition of GO played a significant role in improving the voltage. This could be confirmed from Figure S21 (Supporting Information). After the introduction of an asymmetric GO layer, the output voltage of the A‐RMA/GO@P device with an asymmetric sandwich structure was significantly increased and stabilized at ≈0.544 mV, indicating that the introduced GO layer significantly enhanced the power generation capability of the film. To validate that the continuous power output of the A‐RFA/GO@P device is induced by water evaporation, the following experiment was conducted. First, the A‐RFA/GO@P device was placed in a beaker containing a 3.5 wt.% NaCl solution. When the output voltage was stable at ≈0.55 V, the beaker was sealed with a polyethylene (PE) film to cover the opening. Once sealed, the water vapor inside the beaker quickly reached saturation, and the water evaporation on the surface of the device gradually slowed down and eventually stopped, resulting in the stagnation of evaporation‐induced capillary flow within the film. As shown in Figure 6f, when the beaker was sealed, the output voltage rapidly decreased and gradually approached 0. After opening the beaker, evaporation resumed, leading to an increase in the output voltage of the device. It is worth noting that this process exhibited excellent reproducibility when repeated experiments were conducted, further confirming that the power generation of the A‐RFA/GO@P device is indeed induced by water evaporation. From the perspective of practical application, the ability of continuous and stable operation is an important indicator to consider the reliability of the device. Therefore, the 6 h continuous power generation performance of the A‐RMA/GO@P device was tested, as shown in Figure 6g. One device stably outputs an open circuit voltage of ≈0.544 mV, and the output current was also consistently maintained at ≈13.07 µA under ambient conditions. Ignoring the influence of other factors, the results demonstrated that the A‐RMA/GO@P composite film had the ability to work stably over long periods of time. It was confirmed that the device could continuously output a relatively stable voltage by the test of six‐day power generation (Figure S22, Supporting Information). Furthermore, the effects of different NaCl solution concentrations on the power generation performance were also studied, as shown in Figure S23 (Supporting Information). The dry A‐RMA/GO@P device was almost incapable of generating electrical energy, while in deionized water, the device could output an average voltage of 0.372 V. Moreover, the voltage increased gradually as the NaCl concentration in the solution increased, which was attributed to the monotonic increase in the charge number and the charge carrier (Na^+^) transport gradient in the solution with increasing NaCl content. When the solution salinity increased from 0.1 to 3.5 wt.%, the continuous output voltage could be significantly enhanced from 0.384 to 0.544 mV. Therefore, the output voltage of the device is dependent on the NaCl concentration in the solution.

**Figure 7:**
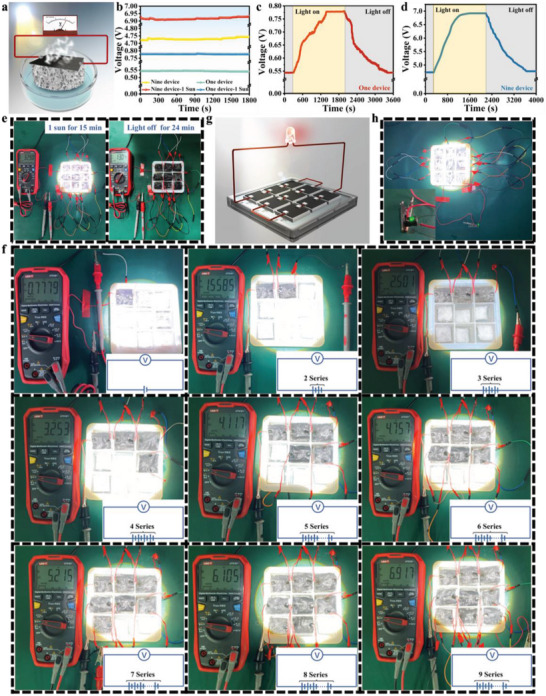
a) Schematic illustration of the A‐RMA/GO@P photothermal evaporator for simultaneous solar desalination and electric power generation under one sun irradiation. b) Comparison of the voltage output with one and nine A‐RMA/GO@P devices under ambient conditions and one sun irradiation. c,d) Voltage output with one device and nine devices under light on and off. e) Current output of nine A‐RMA/GO@P devices before and after light off. f) Voltage output with the A‐RMA/GO@P device by connecting several devices in series (1 device‐9 devices) under one sun irradiation. g) Schematic illustration of the LED lamp lighted up by the nine‐grid integrated array formed by nine devices in series under one sun irradiation. h) Optical picture of the nine‐grid integrated evaporation/generator array lighting LED under one sun irradiation.

To investigate the effect of solar irradiation on the water evaporation‐induced power generation capability of this asymmetric nanofluidic photothermal thin film, an experimental device was designed (**Figure** **7**a). Interestingly, the power generation capacity in the presence of solar irradiation exhibited a significant improvement over the ambient conditions without light. The voltage output performance was tested under sunlight. The voltage generated by the A‐RMA@P and A‐RMA/GO@P devices increased to 0.441 and 0.778 V, respectively (Figure S24, Supporting Information). Under one sun illumination, the voltage output of the single A‐RMA/GO@P device was ≈0.234 V higher than the voltage output under the environmental conditions, as shown in Figure 7b. Similarly, the voltage generated by nine devices in series under one sun illumination could reach ≈6.917 V, but only ≈4.731 V under ambient conditions. Figure 7c,d shows the change in voltage output of one device and nine devices before and after the light switch. As the lamp was lit, the output voltage began to rise and finally reached a stable voltage output. Once the lamp was turned off, the voltage output decreased. This may be because light helped to accelerate the evaporation and transport of water, which expanded the gradient distribution of ions, thereby promoting the transport of ions in micropores and causing high‐performance energy conversion.^[^ ^46^, ^47^ ^]^ The output current performance of nine series devices was tested under solar illumination (Figure 7e). After irradiation for ≈15 min, the current increased to 32.9 µA. When the illumination stopped, the current would gradually decrease. Similarly, the current change results of a single device are shown in Figure S25 (Supporting Information). The output voltage performance of multiple A‐RMA/GO@P devices in series was studied to meet the practical application requirements, as shown in Figure 7f. As the number of devices in the series increased, the output voltage increased monotonically (Figure S26, Supporting Information). In addition, to further validate the operational stability of the nine‐grid array device, it was placed in a saltwater solution with a concentration of 3.5 wt.% for cyclic testing. Under one sun illumination, the water evaporation‐induced power generation performance of the array device was tested at intervals of 0.5 h, and the power generation of the device within 0.5 h was recorded. As shown in Figure S27 (Supporting Information), the results indicate that the average output voltage of the array device was not significantly attenuated and remained above 6.88 V, during ten cycles of testing. This demonstrates its excellent stability and durability, providing essential conditions for its wide application in simultaneous solar seawater desalination and power generation. Figure 7g shows a schematic diagram of the nine‐grid integrated array formed by nine devices in series lighting an LED. Without any additional components, the LED can be successfully lit by the power output of the nine‐grid integrated evaporator/generator under light conditions (Figure 7h). Under ambient conditions, the nine‐grid array can also light the LED, as shown in Figure S28 (Supporting Information).

**Figure 8:**
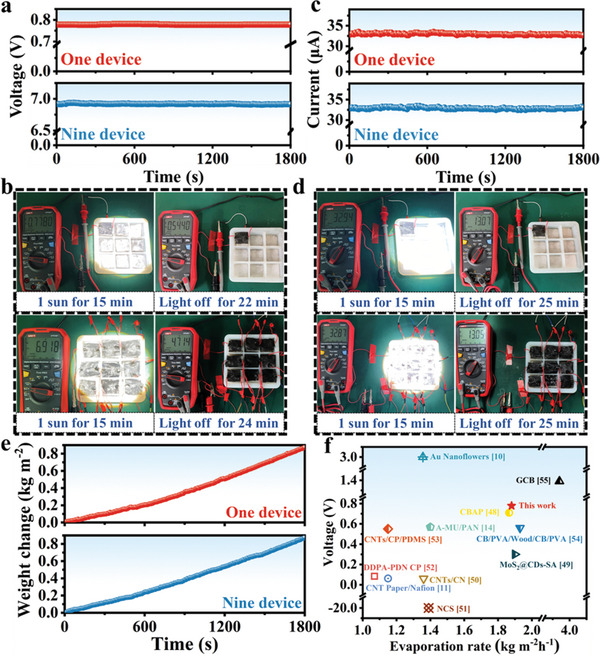
a–e) Simultaneous performance of solar evaporation and energy generation of one and nine A‐RMA/GO@P devices under one sun irradiation. f) Performance comparison of our device to some recent reports.

The performance of the A‐RMA/GO@P devices was studied for simultaneous evaporation and electricity production under sunlight. Under one sun, the average output voltage of the single A‐RMA/GO@P device was ≈0.778 V, while the output voltage of the nine devices could be as high as ≈6.918 V, as shown in **Figure** **8**a,b. Moreover, the output current could rise to 32.94 and 32.87 µA for single and nine devices, respectively, after 15 min of one sun irradiation (Figure 8c,d). Both single and nine A‐RMA/GO@P devices generating high power output also maintained a stable evaporation rate, showing an evaporation rate of ≈1.74 kg m^−2^ h^−1^, as shown in Figure 8e. The above results indicate that the A‐RMA/GO@P device prepared by the asymmetric load can be used as an efficient solar evaporator, and the electrical energy will be collected from the water evaporation process under light/dark conditions around the clock. The overall design achieves the effective utilization of the water evaporation process to generate both electrical energy and clean water, which can maximize the use of environmental resources and amplify the output of electrical energy through a series of multiple devices to ensure practical application requirements.

Furthermore, as shown in Figure 8f, our device is among the top integrated photothermal evaporator/nanogenerators for synergistic steam generation and electricity production with better performance in terms of both solar evaporation rate and power generation capacity compared to previously reported synchronous solar steam generators.^[^ ^10^, ^11^, ^14^, ^48^, ^49^, ^50^, ^51^, ^52^, ^53^, ^54^, ^55^ ^]^ More importantly, we demonstrate a scalable strategy for the practical applications of solar evaporation and power generation. The electrical energy generated by evaporation is more reasonably and effectively utilized. The total evaporation area and power output are improved through the series connection of multiple devices. Moreover, a nine‐grid device array containing nine devices is designed, which integrates solar water evaporation and full‐time power generation into one device. The nine‐grid device array achieves a competitively high evaporation rate and power output, showing the practical application value of powering devices directly. Our designed RMA‐based evaporator for synergistic photothermal evaporation and water evaporation‐induced power generation has good application prospects, which provides a new method for the conversion and comprehensive utilization of solar energy.

## 3 Conclusion

In summary, we have developed a multifunctional solar interface evaporator through electrokinetic effect‐induced power generation, which enables the simultaneous generation of fresh water and electricity. The device can achieve a high evaporation rate of 1.88 kg m^−2^ h^−1^ and solar photothermal conversion efficiency of 94.3% due to the good synergy of the RMA composites and the excellent thermal management properties of the 3D aerogel. More importantly, we designed and demonstrated an RMA‐based asymmetric strategy to prepare a nonuniform sandwich structure film by adding GO layers to multidimensionally expand the asymmetry of the film. The evaporation rate and average voltage output of the asymmetric RMA/GO@air‐laid paper (A‐RMA/GO@P‐SA) evaporator that can work around the clock can be maintained at 1.74 kg m^−2^ h^−1^ and 0.778 V, respectively, under one sun irradiation. The device can continue to operate under ambient conditions with an electric energy output of ≈0.554 V and 13.07 µA, showing excellent stability, durability, and versatility. In addition, the nine‐grid integrated array assembled by nine devices in series can improve the evaporation area and power production capacity. The output voltage is as high as 6.917 V and an LED lamp can be lit. The nine‐grid array with the advantages of small size, easy portability, and low environmental impact has the potential for large‐scale production and application. This work may provide a new method for long‐term sustainable freshwater harvesting and power generation with stable power output.

## 4 Experimental Section

### Materials and Chemicals

GO was supplied by Suzhou TANFENG Graphene Tech Co., Ltd. (Suzhou, China). Manganese chloride tetrahydrate (MnCl_2 _· 4H_2_O), sodium alginate (SA), and gluconate lactone were obtained from Macklin Biochemical Co., Ltd (Shanghai, China). Aluminum powder was provided by Aladdin Bio‐Chem Technology Co., Ltd. (Shanghai, China). Ammonium hydroxide and calcium carbonate were obtained from Beijing Tongguang Fine Chemical Co., Ltd. (Beijing, China).

### Preparation of RGO/Mn_3_O_4_‐Based Composite Film

First, NH_3_·H_2_O solution (3 mL) was added to the GO dispersion (6 mg mL^−1^) to obtain an alkaline dispersion under magnetic stirring. Then, MnCl_2_·4H_2_O (660 mg) was stirred in the dispersion (60 mL) for 2 h. Subsequently, aluminum powder (100 mg) was mixed with the solution and allowed to stand for 7 h at room temperature. Then, the product was washed and centrifuged five times and dried at 60 °C for 12 h. Finally, the composites (100 mg) were ultrasonically dispersed in deionized (DI) water (30 mL), and air‐laid paper (3 cm × 3 cm) was immersed in the solution for 20 min and dried at 60 °C. After three repetitions, the RGO/Mn_3_O_4_/Al_2_O_3_‐air‐laid paper (RMA‐P) membrane was obtained. The same process was used for synthesizing the RGO/Mn_3_O_4_‐air‐laid paper (RM‐P) film without the addition of aluminum powder.

### Preparation of 3D Aerogel

Sodium alginate (SA 500 mg) and calcium carbonate (500 mg) were added to DI water (30 mL) and magnetically stirred for 1 h. After that, gluconate lactone (800 mg) was mixed and the mixture was left to stand for 5 min, followed by vertical freezing for 12 h. Finally, 3D aerogel could be obtained by drying it for 48 h in a freeze‐dryer.

### Characterization

Scanning electron microscopy (SEM; Regulus 8100, Hitachi) and transmission electron microscopy (TEM; HT7800, Hitachi) were used to observe the surface topography and microstructure of the RM, RMA, air‐laid paper, RMA‐P, and SA aerogel. X‐ray photoelectron spectroscopy (XPS) experiments of GO and RMA were conducted on a PHI QUANTERA‐II SXM. The functional groups of the composite materials were analyzed by a Fourier transform infrared (FT‒IR) spectrometer (NICOLET iS50, USA). A LabRAM HR 800UV (HORIBA Jobin Yvon, France) with an operating wavelength of 532 nm was used for recording Raman spectra of composite materials. X‐ray diffraction (XRD; Bruker, Germany) was used to reveal the crystal structure from 10° to 80°. The optical absorption performance was recorded by UV−vis−NIR spectrophotometry (UV‒vis‒NIR; LAMBDA 1050, Japan). The zeta potential was measured by a zeta potential instrument (Zetasizer Nano ZS ZEN3600, Malvern Instruments, UK). A multimeter (UNI‐T‐UT61E+) was used to measure the voltage and current generated by water evaporation.

### Preparation of Water‐Driven Energy Generation

RGO/Mn_3_O_4_/Al_2_O_3_‐air‐laid paper (RMA‐P) was synthesized by the solution‐loading method. The RMA composites (100 mg) were ultrasonically dispersed in 30 mL of deionized water. The air‐laid paper was immersed vertically in the well‐dispersed solution to form an asymmetric distribution of less upper‐load materials and lower‐load materials and then dried at 60 °C. After repeating the process three times, nonuniformly distributed RMA‐P composite films were successfully acquired. GO@P and RM@P composite films were obtained by the same operation. To obtain nonuniform RMA/GO@P, the air‐laid paper was vertically dipped in the RMA suspension, GO suspension (1.5 mg mL^−1^), and RMA suspension, successively. After three drying cycles, the asymmetric RMA/GO@P composite films were successfully prepared. The conductive silver adhesive coated on both ends of the film could connect the copper wires to the film and act as conductive electrodes. The copper wires and electrodes were sealed with epoxy resin, which served to avoid unwanted corrosion and potential electrochemical reactions. A porous hydrophilic and well‐insulated SA aerogel was placed under the evaporator, which was used for the support layer and water transport channels.

### Solar Evaporation Test

A solar simulator (Solar‐500, Beijing NBeT Technology Co., Ltd., Beijing, China) with a standard spectrum filter (AM 1.5) was used for executing the solar evaporation test. The intensity of solar irradiation was calibrated to one sun illumination (1 kW m^−2^) at the sample level by a full‐spectrum optical power meter (Beijing NBeT Technology Co., Ltd., Beijing, China). A computer connected to an electronic balance (PTX‐FA210s) with an accuracy of 0.1 mg was used for recording the real‐time weight changes of different samples during the overall duration of the steam generation experiments. The surface temperature distribution was measured by an infrared camera (Fluke Ti32, Fluke Electronic Instrument Co., Ltd., USA). The surface temperature data were investigated by a thermocouple (TASI, TA612C, China). The entire solar evaporation and electricity generation experiments were conducted at a room temperature of 26–28 °C and a humidity of 40–45 RH%.

## Conflict of Interest

The authors declare no conflict of interest.

## Supporting information

Supporting Information

## Data Availability

The data that support the findings of this study are available from the corresponding author upon reasonable request.
