# Supplementary material for: Achieving Solar‐Thermal‐Electro Integration Evaporator Nine‐Grid Array with Asymmetric Strategy for Simultaneous Harvesting Clean Water and Electricity
Source: Adv Sci (Weinh). 2023 Sep 22;10(31):2303815. doi: 10.1002/advs.202303815 (PMC10625061; doi:10.1002/advs.202303815)
Supplement: Supplementary file 1 — Supporting Information [file ADVS-10-2303815-s001.pdf]

ADVANCED SCIENCE
Open Access

Supporting Information

for *Adv. Sci.*, DOI 10.1002/advs.202303815

Achieving Solar-Thermal-Electro Integration Evaporator Nine-Grid Array with Asymmetric Strategy for Simultaneous Harvesting Clean Water and Electricity

Junli Ma, Zhenzhen Guo, Xu Han, Heng Lu, Kaixin Guo, Jianguo Xin*, Chaoyong Deng* and Xianbao Wang**

Supporting Information

Achieving Solar-Thermal-Electro Integration Evaporator Nine-Grid Array with Asymmetric Strategy for Simultaneous Harvesting Clean Water and Electricity

Junli Ma, Zhenzhen Guo, Xu Han, Heng Lu, Kaixin Guo, Jianguo Xin*, Chaoyong Deng*, Xianbao Wang*

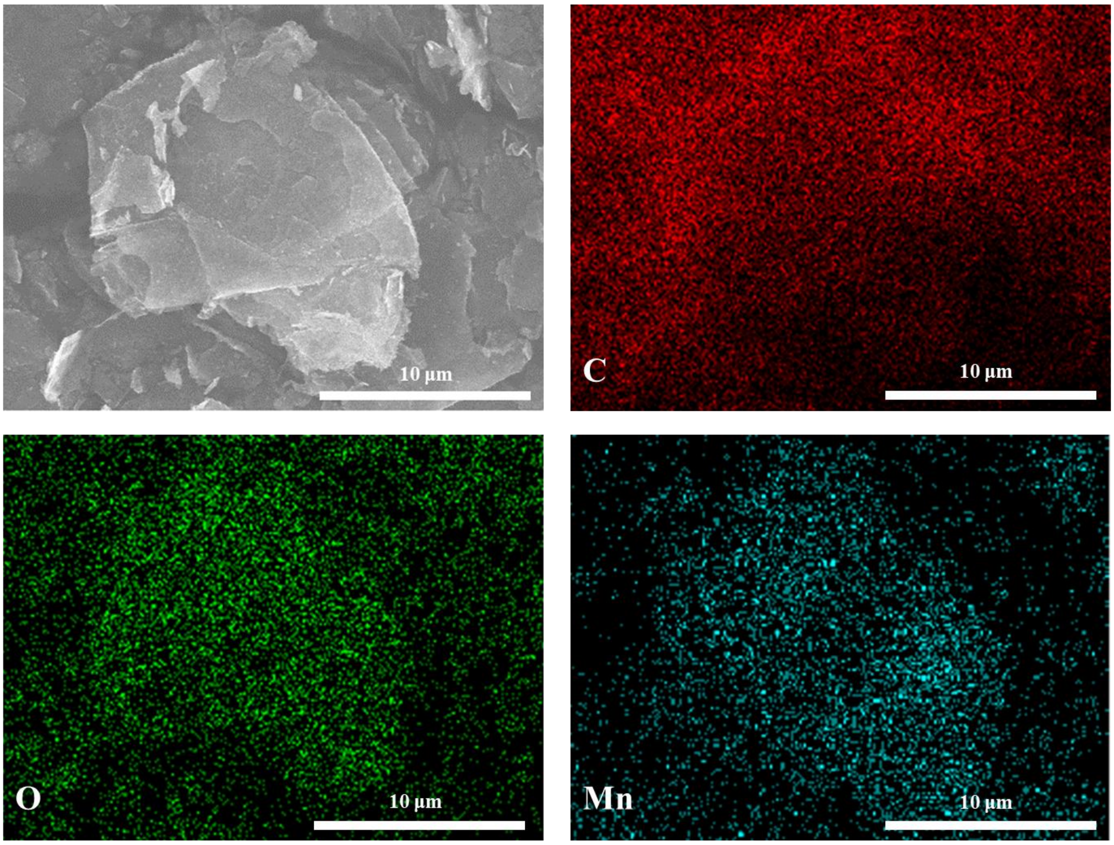

Figure S1. SEM image and corresponding elemental mapping images of C, Mn, O and Al elements of RM.

Element	Weight%	Atomic%
C K	68.60	76.52
O K	26.59	22.27
Mn K	4.80	1.21
Totals	100.00	

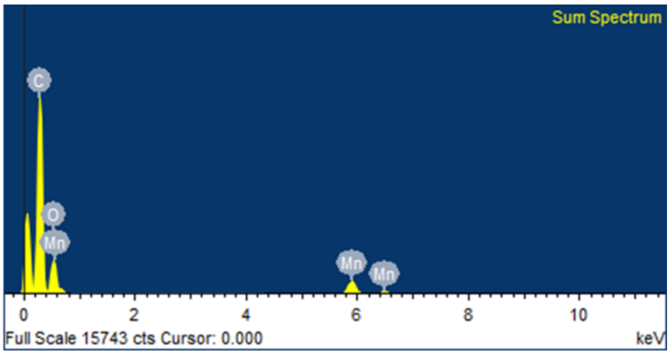

Element	Weight%	Atomic%
C K	53.35	62.35
O K	39.68	34.82
Al K	3.96	2.06
Mn K	3.01	0.77
Totals	100.00	

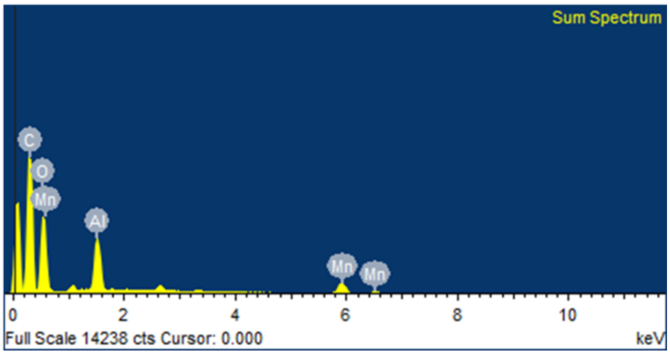

Figure S2. EDS patterns of RM@P and RMA@P films.

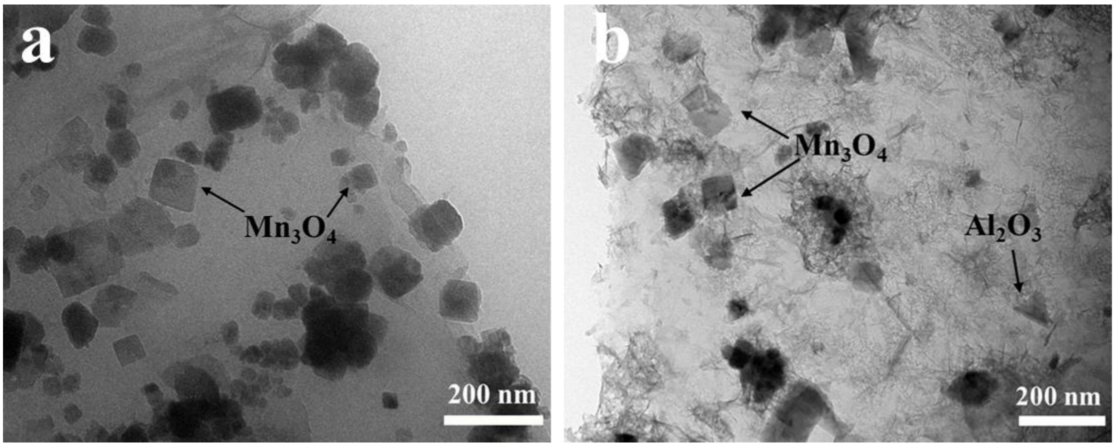

Figure S3. (a, b) TEM images of RM and RMA.

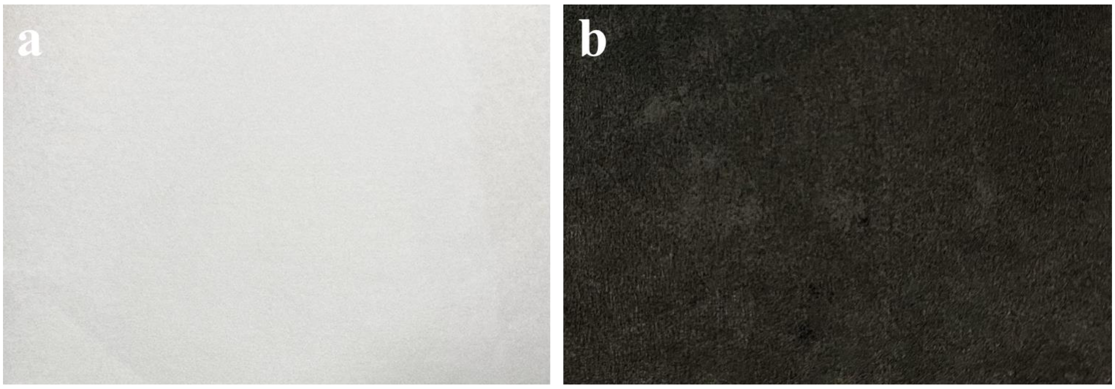

Figure S4. (a, b) Optical picture of the blank film and the RMA@P film.

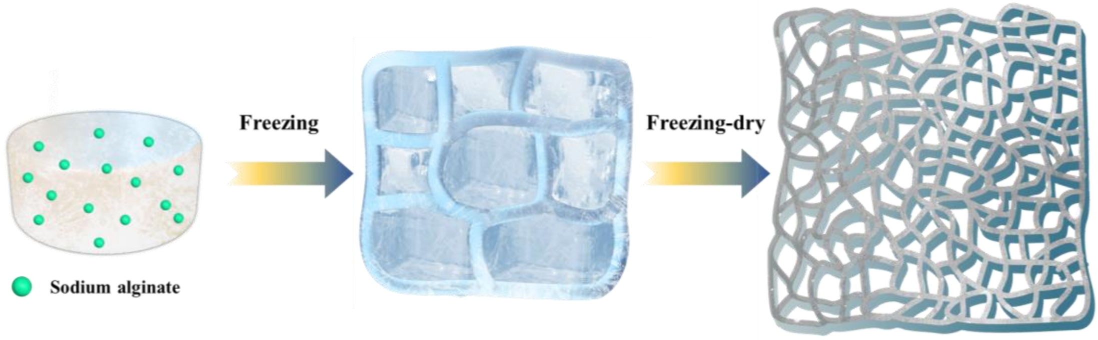

Figure S5. The preparation process of SA.

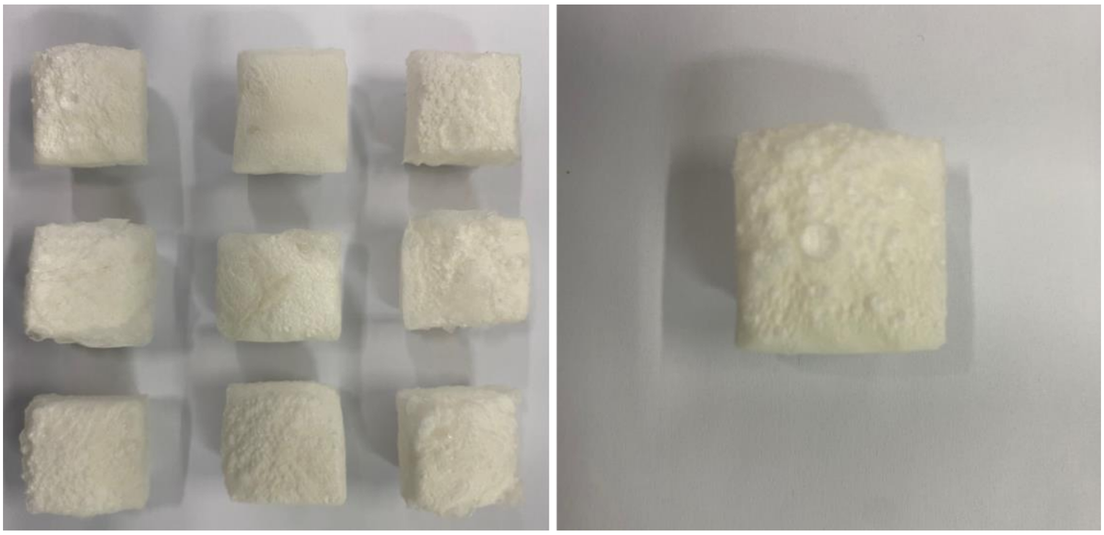

Figure S6. Optical picture of the 3D aerogels.

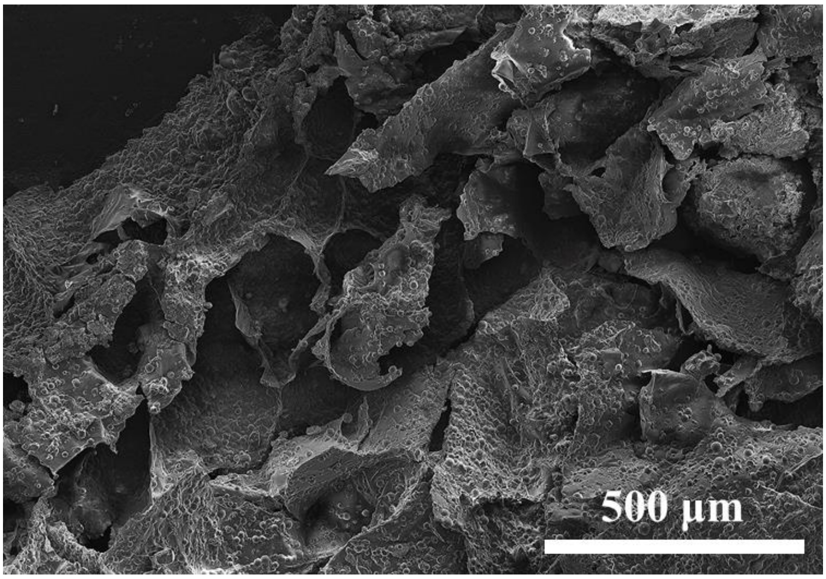

Figure S7. SEM images of the 3D aerogels.

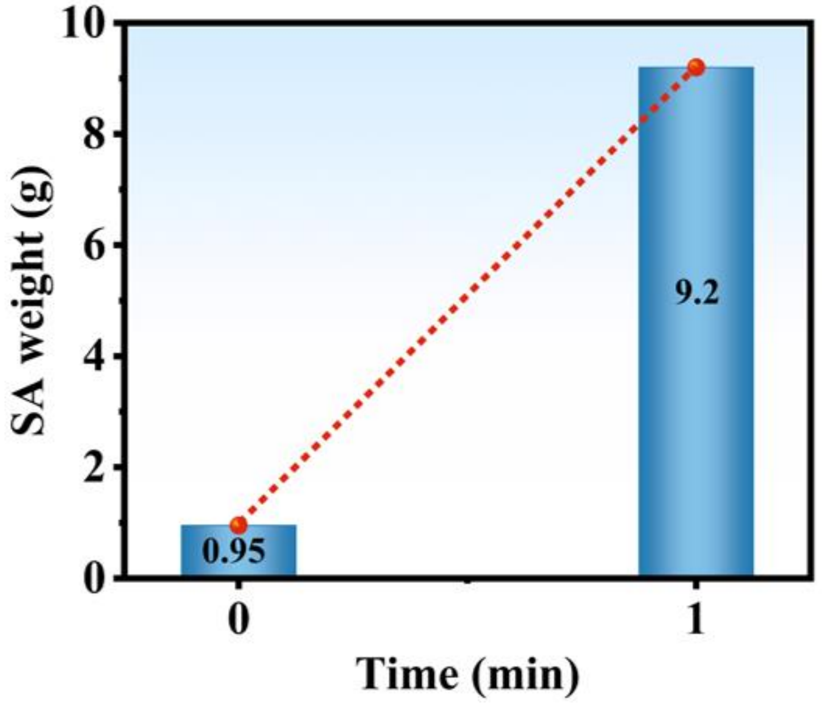

Figure S8. Mass change of SA aerogel absorbing water within 1 min.

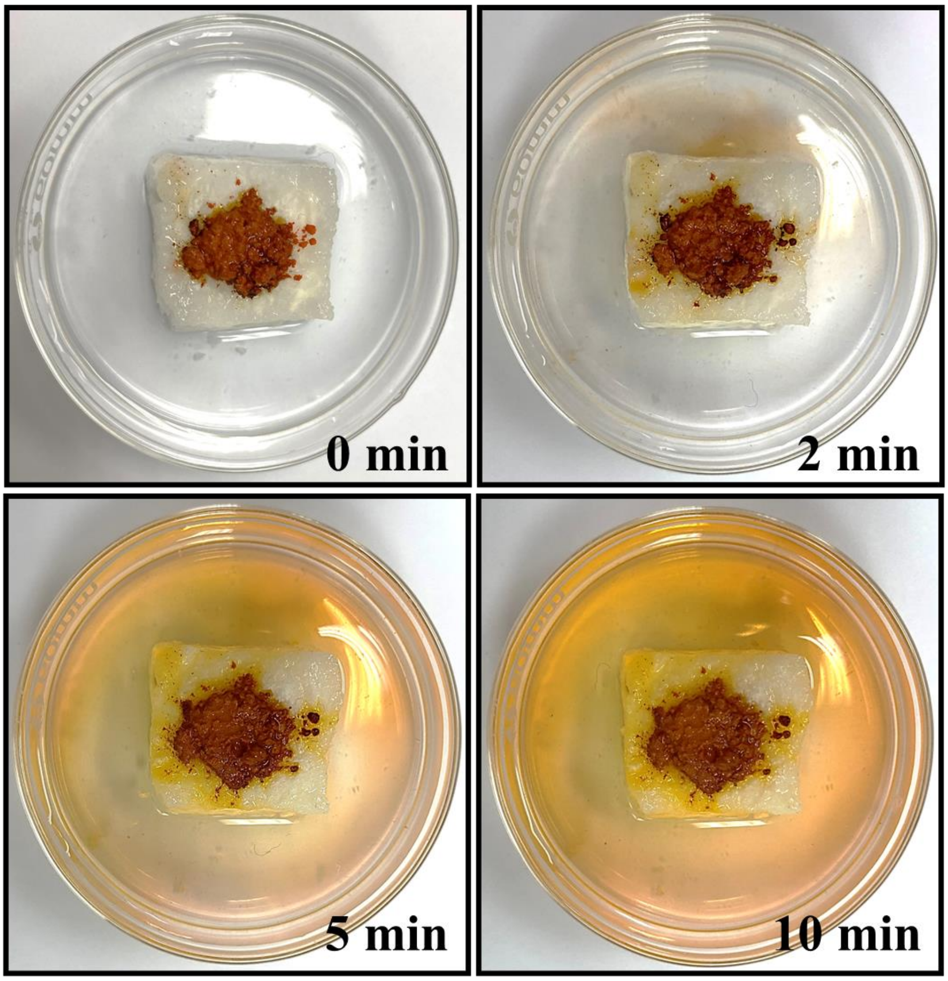

Figure S9. The water supply capacity test of SA aerogels: with the change of time, the MO is dissolved by the water through the evaporator.

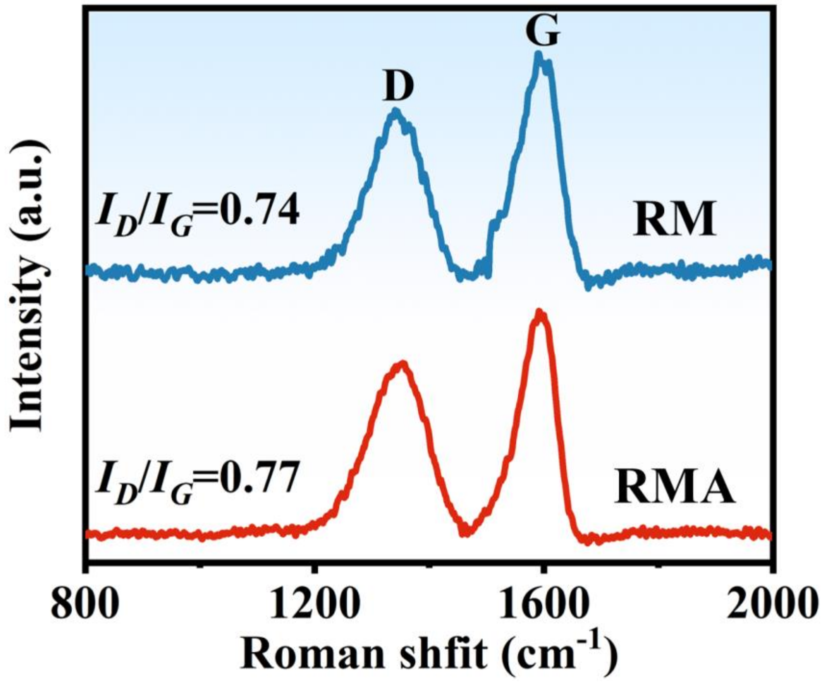

Figure S10. Raman spectra of RM and RMA.

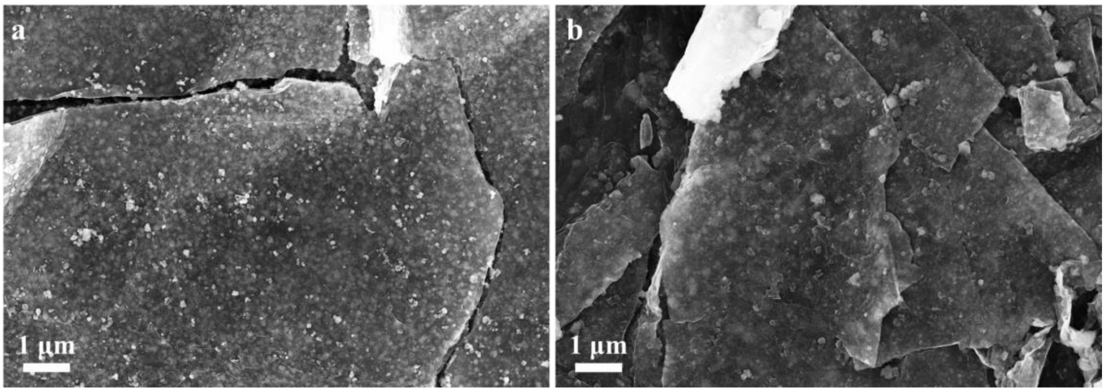

Figure S11. (a, b) SEM images of RM and RMA.

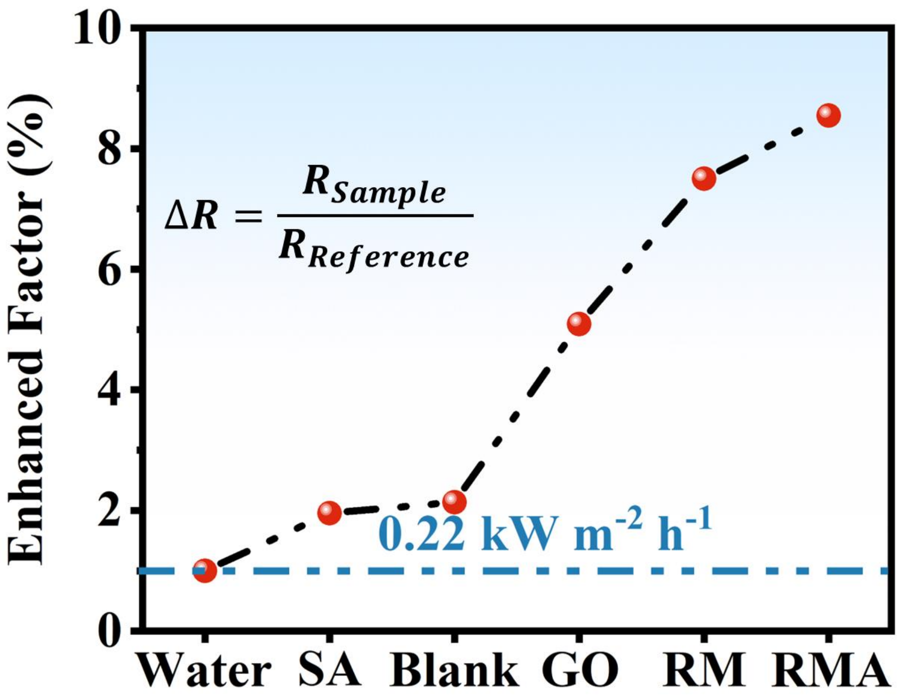

Figure S12. Enhancement Factor (i.e. ΔR) of different devices under 1 sun.

Calculation S1. Photothermal conversion efficiency.[1-3]

$$H_{\mathrm{LV}} = \Delta H + C \times \Delta T \qquad (1)$$

$$\Delta H = 1.91846 \times 10^{3} \times \left(\frac{T_v + 273}{T_v + 239.09}\right)^2 + C \times \Delta T \qquad (2)$$

$$\Delta T = T_v - T_0 \qquad (3)$$

where ΔH is the potential evaporation enthalpy of water, which is related to the temperature of the vapor. C is the specific heat capacity of water (4.2 kJ kg^{-1} K^{-1}), ΔT is the difference between the temperature of vapor (T_v) and the initial temperature of the evaporated liquid (T_0=25 ℃).

Calculation S2. The heat loss of evaporation process containing three types of heat loss that occur during the steam generation, radiation loss, convection loss and conduction loss.[4-6]

(1) The radiation loss was calculated by Stefan-Boltzmann law.

$$\eta_{\mathrm{radiation}} = \frac{q_{radiation}}{q_{solar}} = \frac{\sigma\varepsilon}{q_{solar}}\left(T_s^{\,4} - T_e^{\,4}\right) \qquad (4)$$

where ε represents the emissivity, ε is 1 for RGO/Mn_3O_4/Al_2O_3 composite film, which is supposed the maximum emissivity in this equation. σ is the Stefan-Boltzmann constant equaling to 5.67 × 10^{-8} W m^{-2} K^{-4}. The infrared thermal imager is applied to the surface temperature of different materials, T_s is the average surfance

temperature (42.2 °C) of RGO/Mn_3O_4/Al_2O_3 composite film at a steady state condition, T_e is the ambient temperature(29.9 °C) upward the absorber under one sun, and q_{solar} is used to represent illumination intensity . The radiative loss is ~8.2%.

(2) Newton's law was used to calculate the convective loss.

$$\eta_{convection} = \frac{q_{convection}}{q_{solar}} = \frac{h}{q_{solar}}\left(T_s - T_e\right) \qquad (5)$$

Where h is the the convective heat transfer coefficient, which is about 0.92 W m^{-2} K^{-1}. The convective loss is ~1.1%.

The conductive loss was calculated as following.

$$\eta_{conduction} = \frac{E_{conduction}}{E_{solar}} = \frac{cm\Delta T}{Aq_{solar}t} \qquad (6)$$

where C is the specific heat capacity of water, m is the weight loss of the bulk water used in this experiment, ΔT is the temperature change of the bulk water, A represents the surface area, and t is the radiation time. C is 4.2 J g^{-1} K^{-1}. ΔT is 0.6 °C. A is 28.26 cm^2, m is 50 g, and t is 1800 s. The conduction loss is ~2.5%.

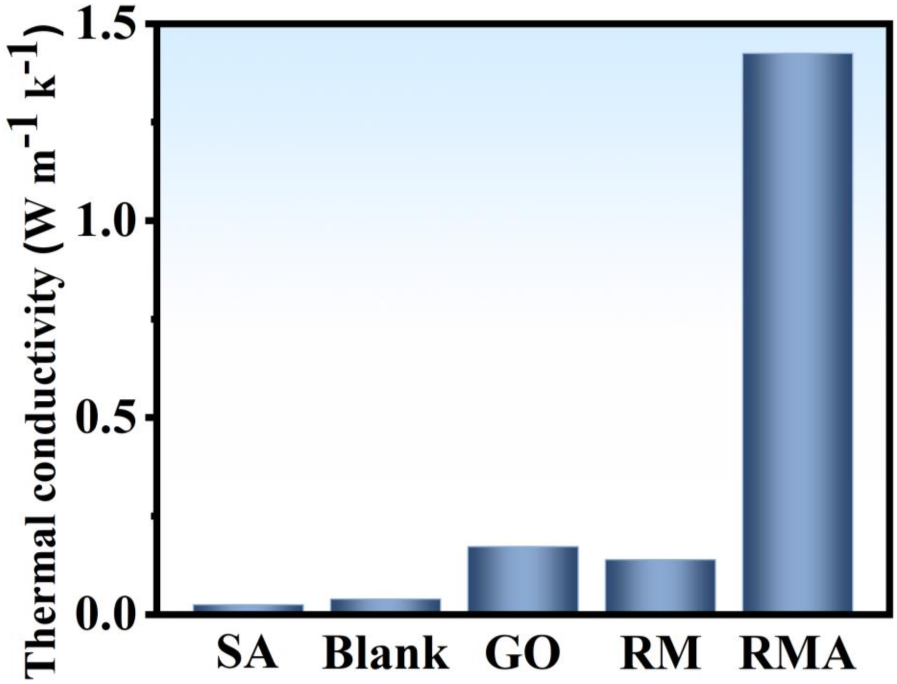

Figure S13. Thermal conductivity of different composite materials.

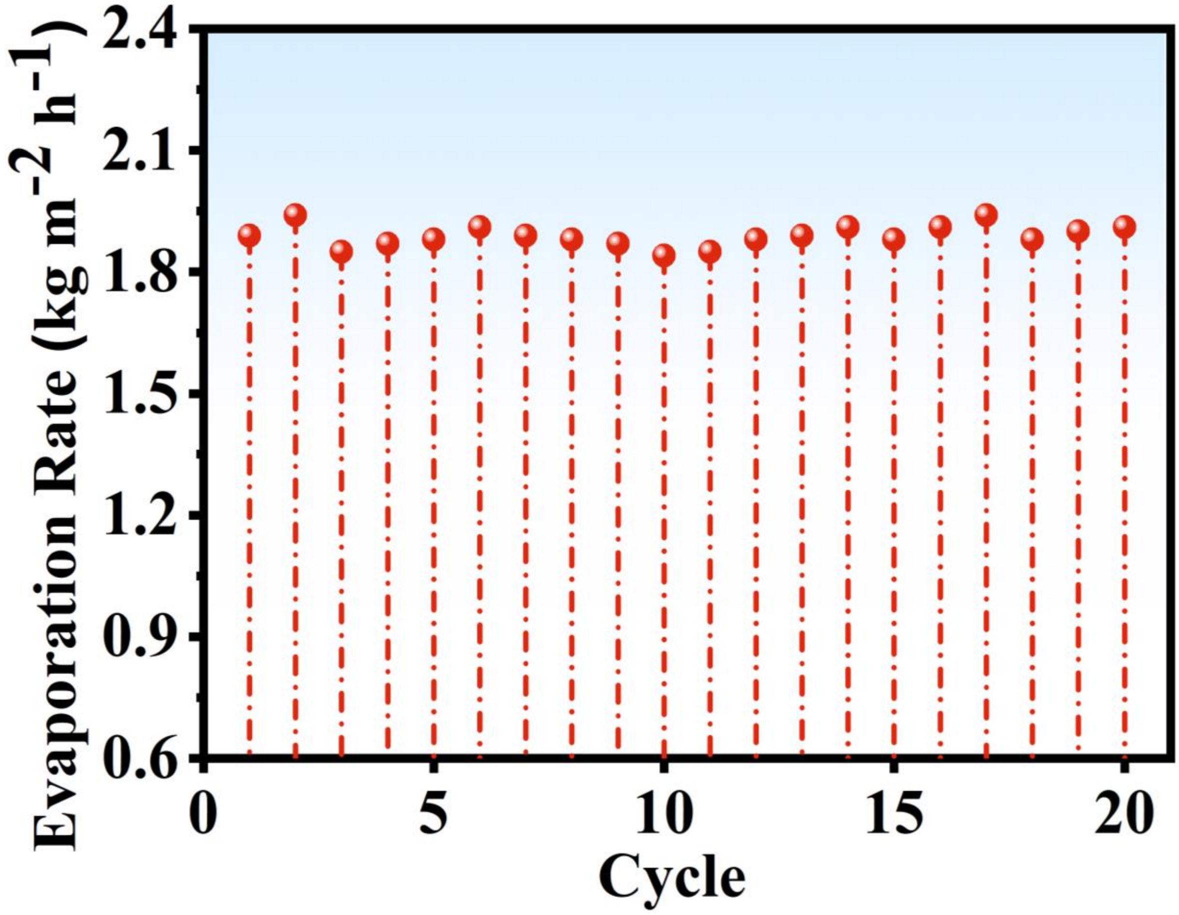

Figure S14. RMA@P-SA evaporation system cycle test.

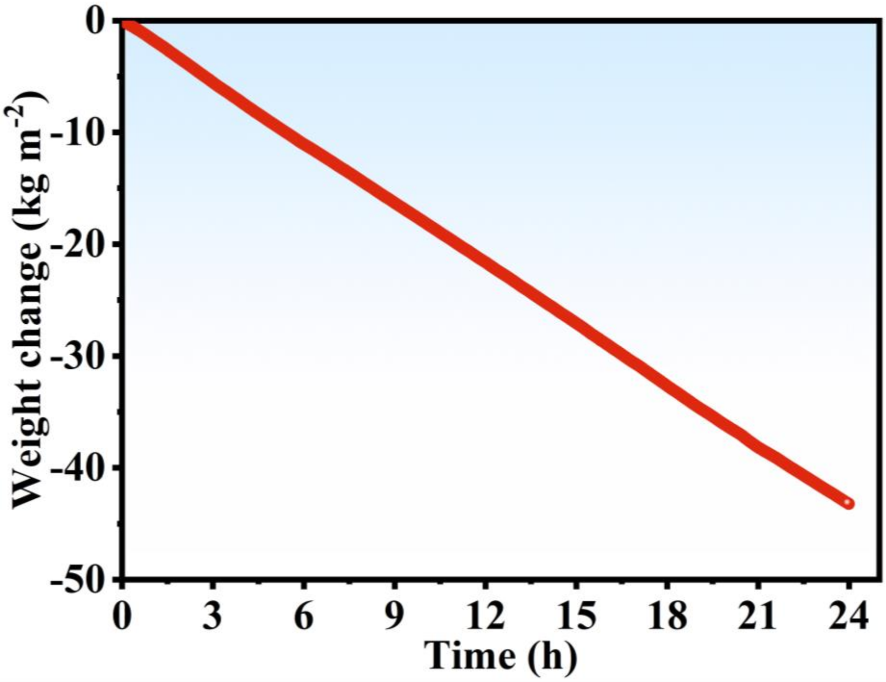

Figure S15. The evaporation performance of high-concentration brine (10 wt% NaCl).

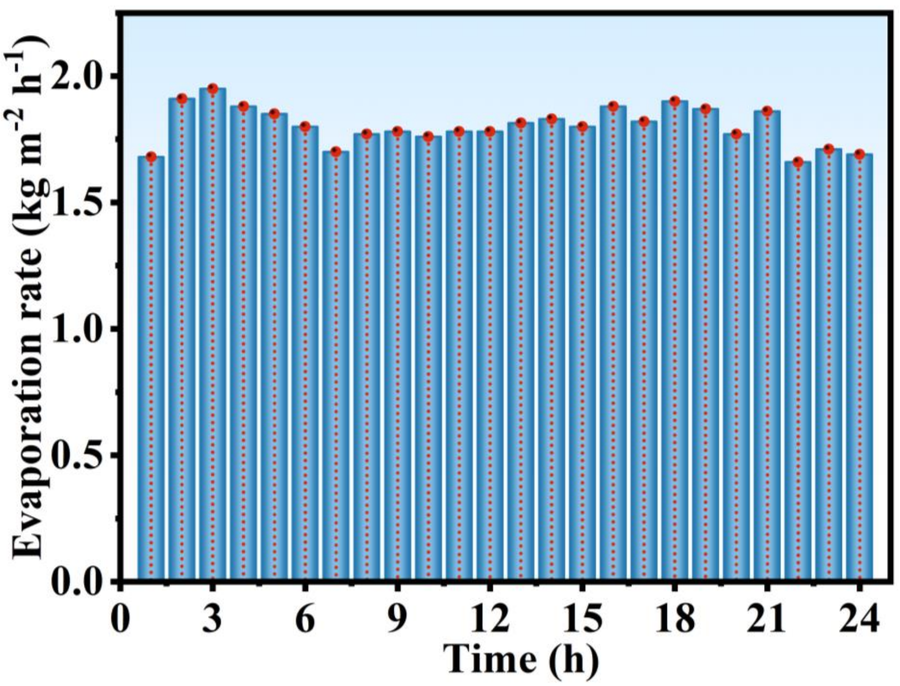

Figure S16. The evaporation rate of RMA@P-SA.

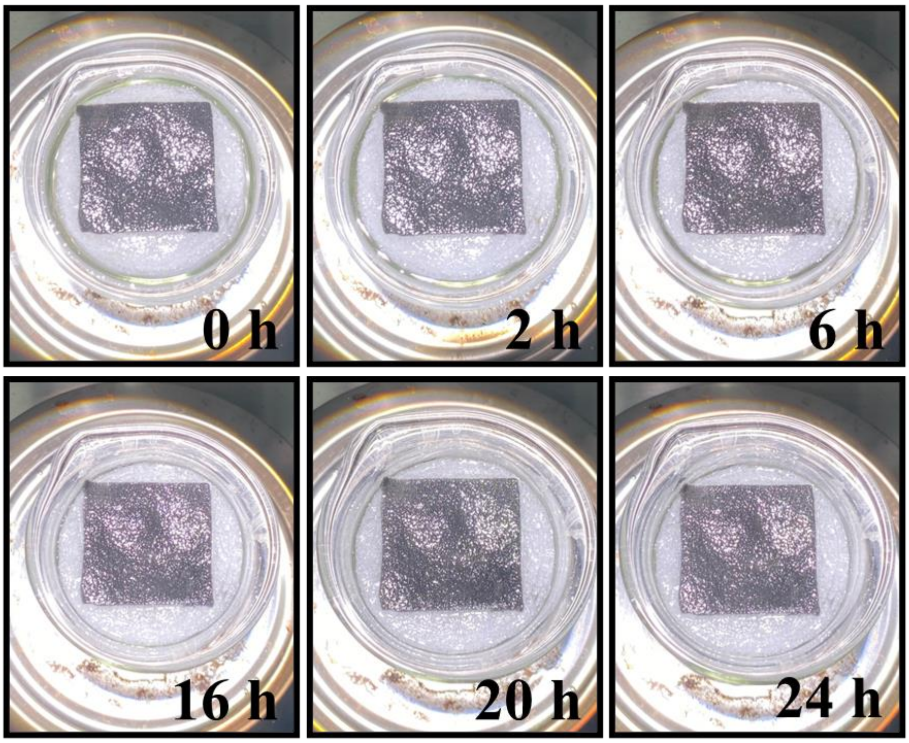

Figure S17. Digital photographs of the RMA@P-SA during continuous operation (one sun).

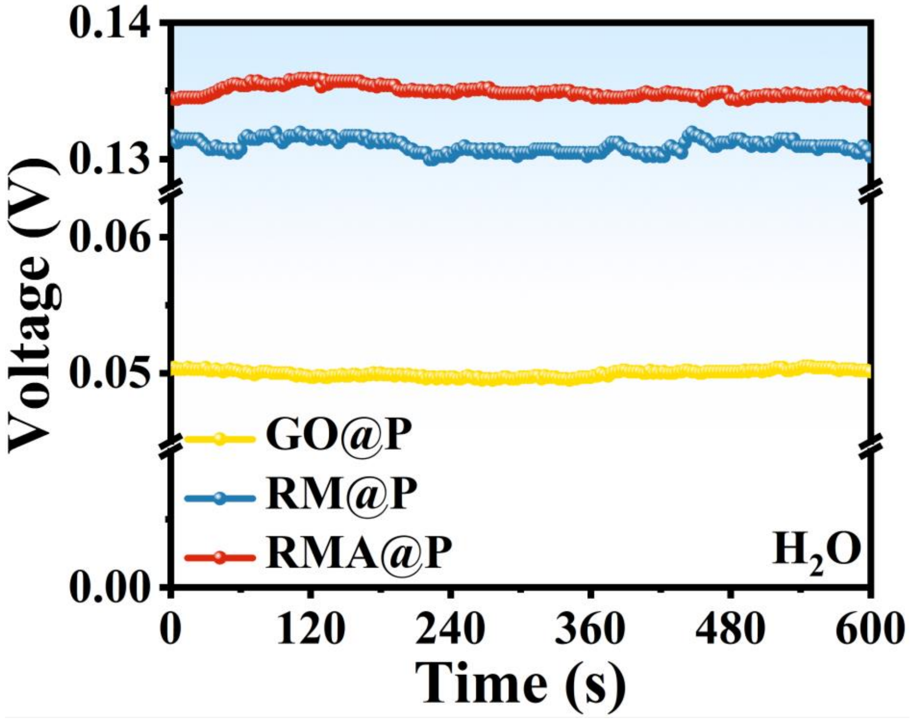

Figure S18. Voltage output from GO@P, RM@P and RMA@P generator immersed by water under ambient conditions.

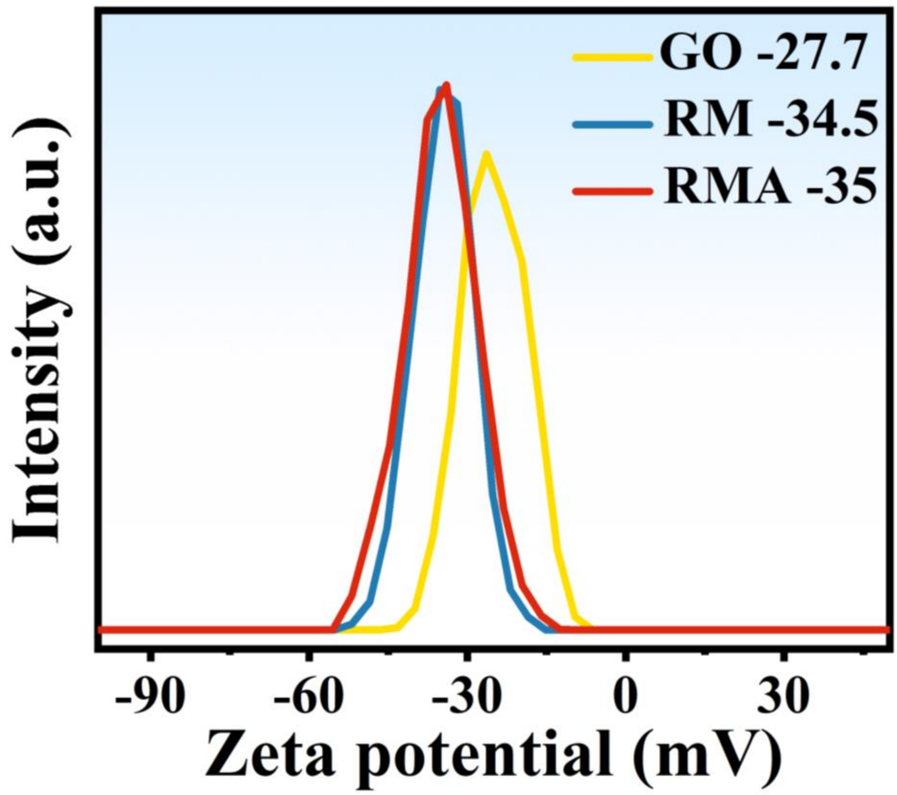

Figure S19. Zeta potential of GO, RM, RMA composites.

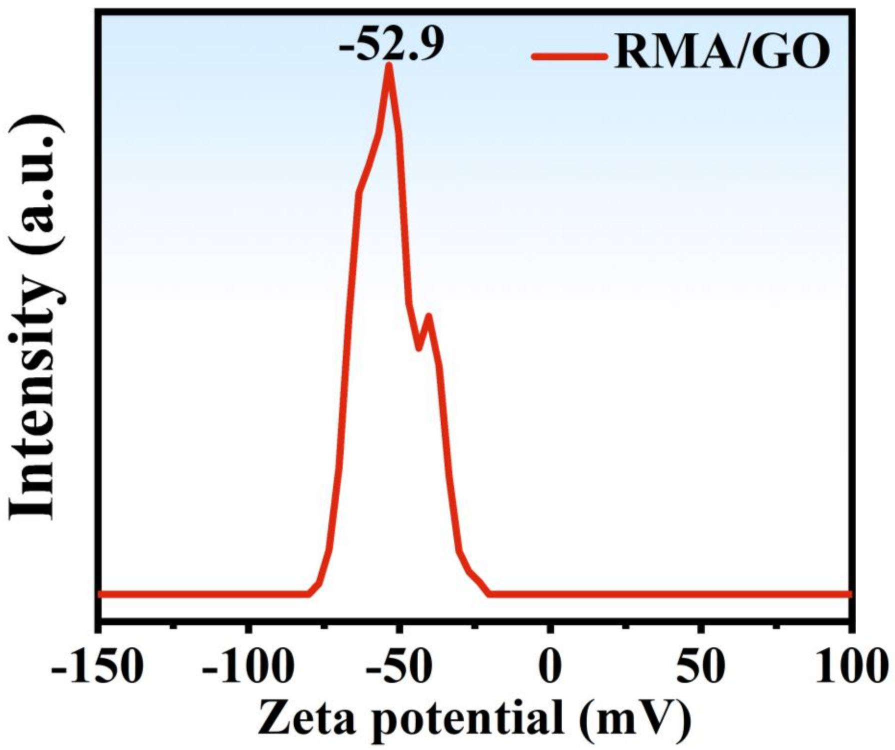

Figure S20. Zeta potential of RMA and GO (mass ratio 2:1) composites.

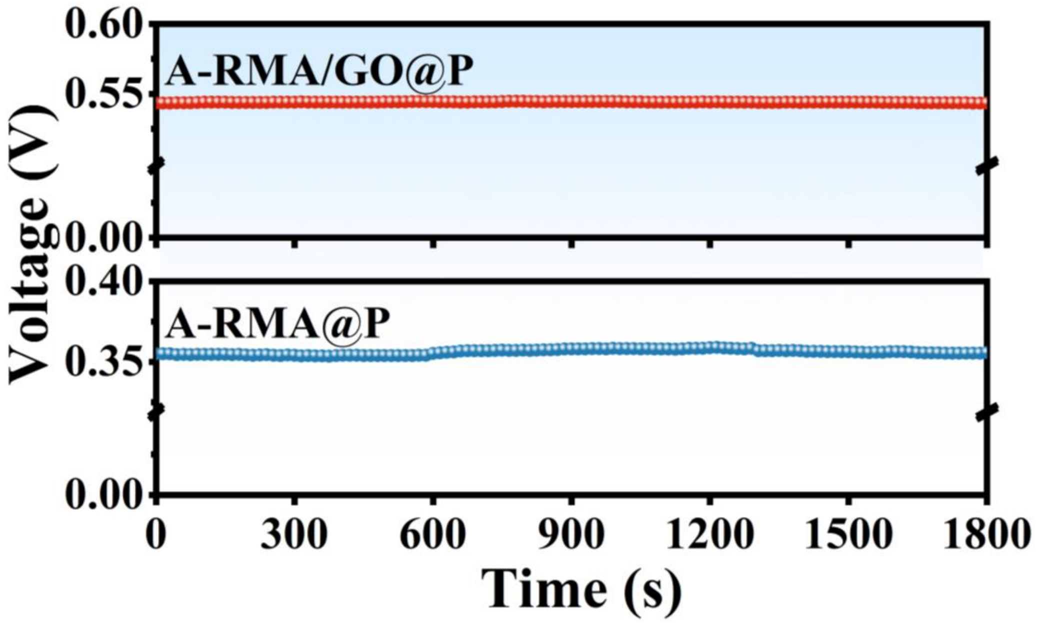

Figure S21. Voltage output from different generators under ambient conditions.

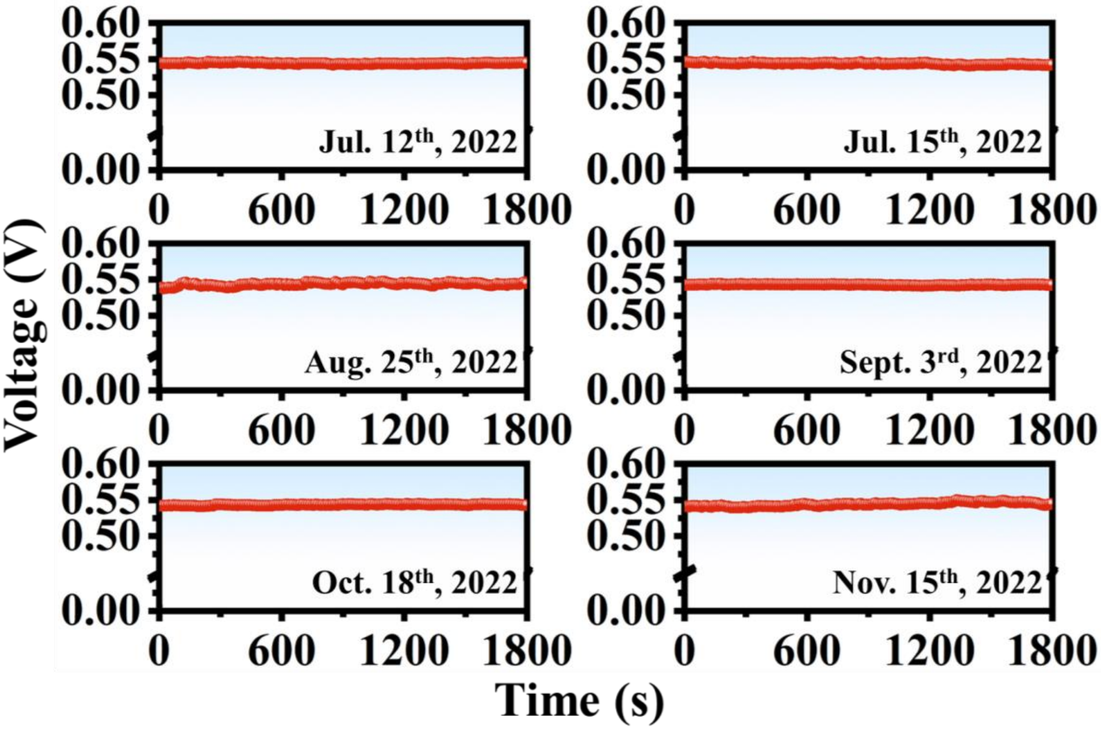

Figure S22. Performance of water-induced energy generation for A-RMA/GO@P device on different dates under ambient conditions.

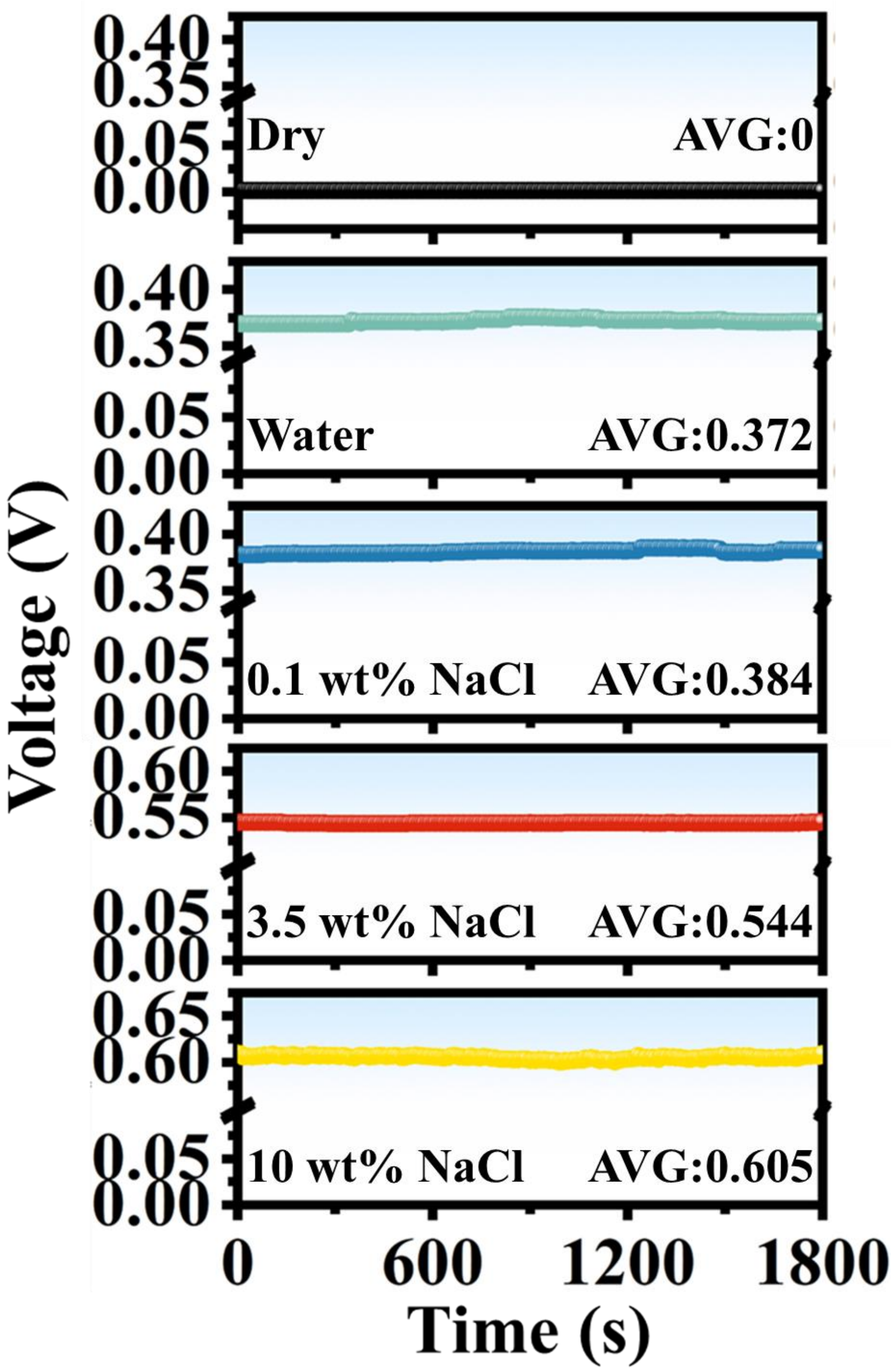

Figure S23. Output voltage of the A-RMA/GO@P device in NaCl solution with different concentrations.

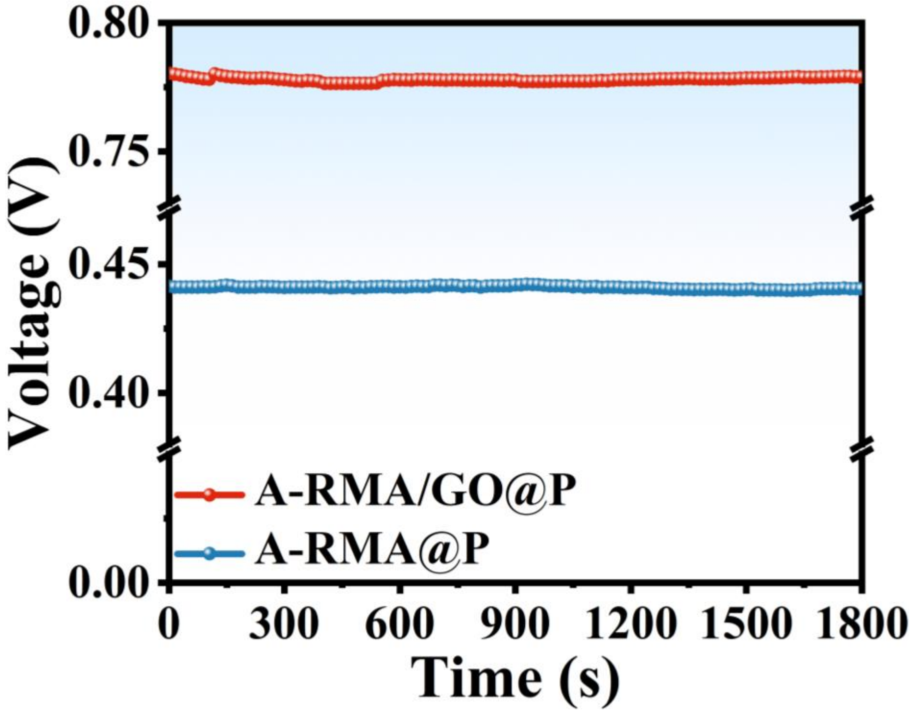

Figure S24. Comparison of the voltage output with A-RMA @P and A-RMA/GO@P device under one sun irradiation.

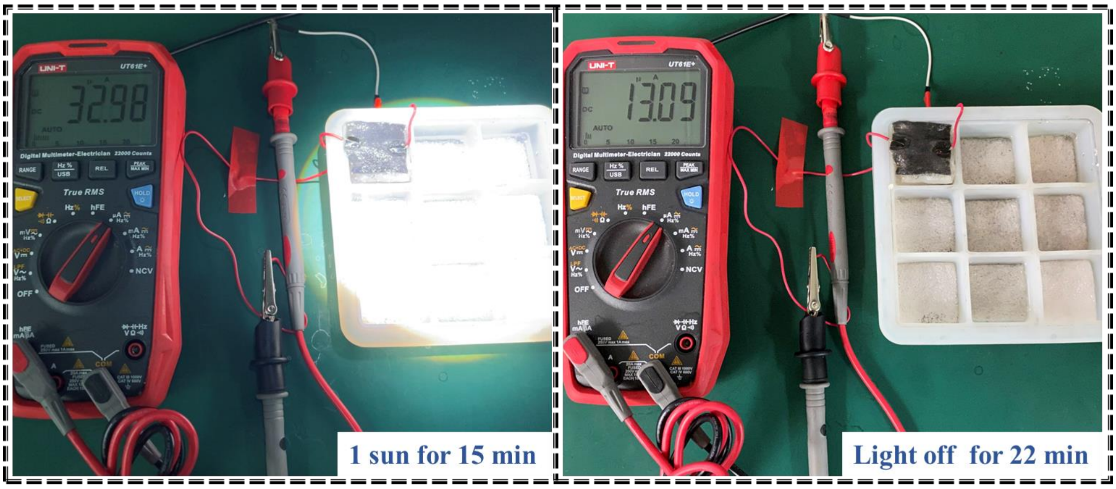

Figure S25. Current output of one A-RMA/GO@P device before and after light off.

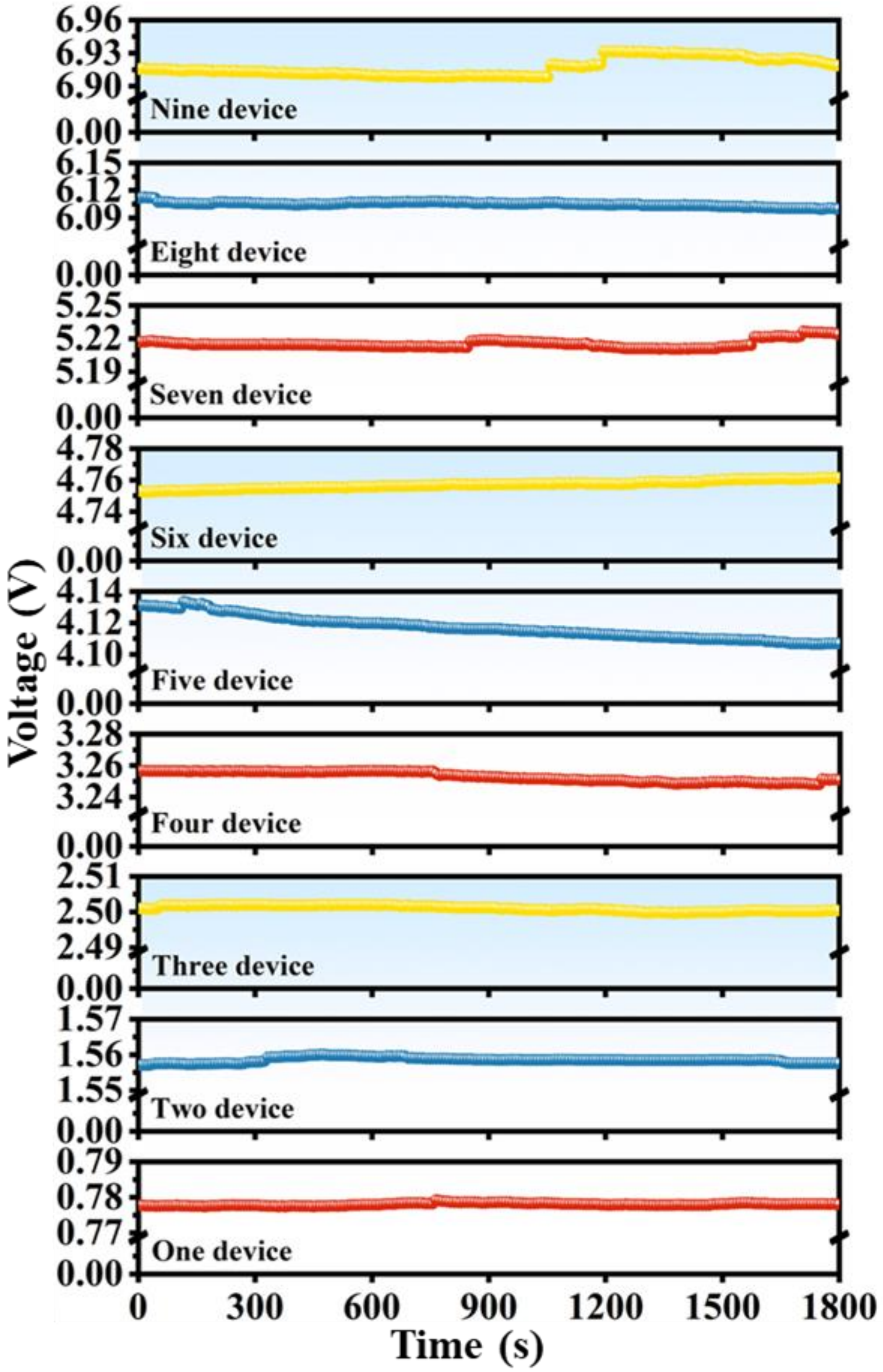

Figure S26. Voltage output of A-RMA/GO@P device by connecting several devices in series (1 device-9 devices) under one sun irradiation.

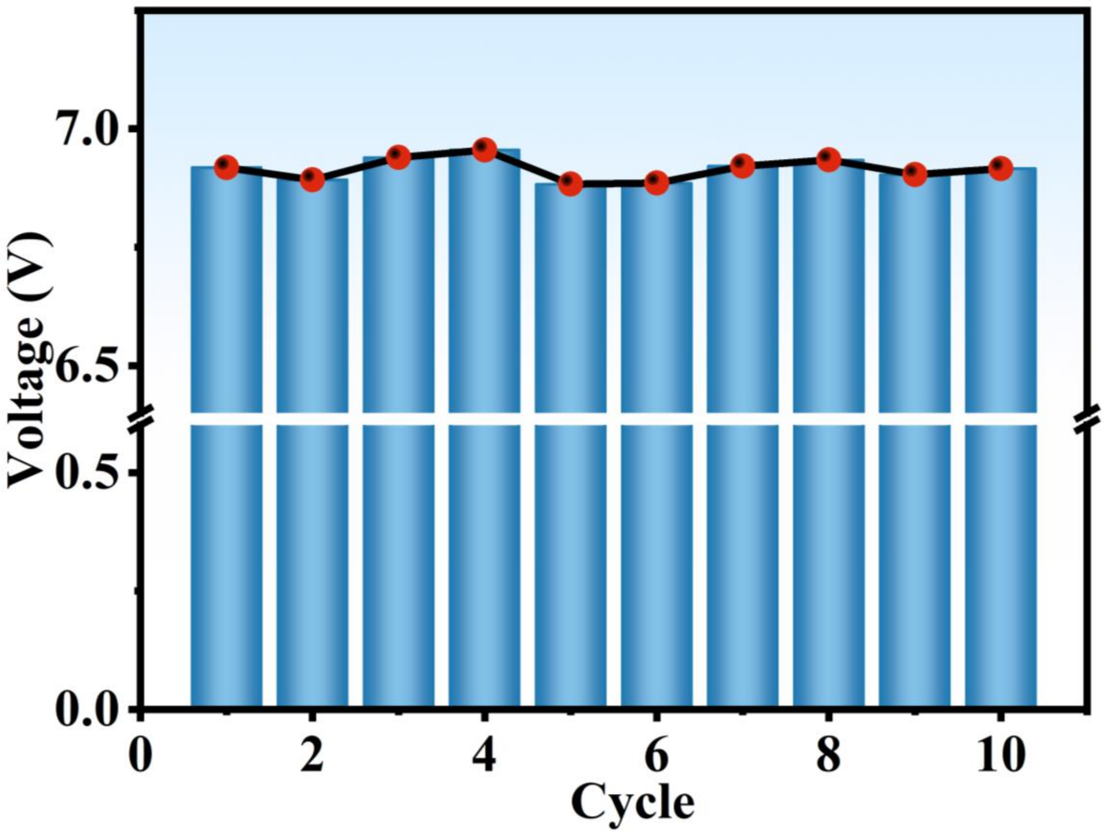

Figure S27. The nine-grid integrated evaporation/generator cycle test under one sun irradiation.

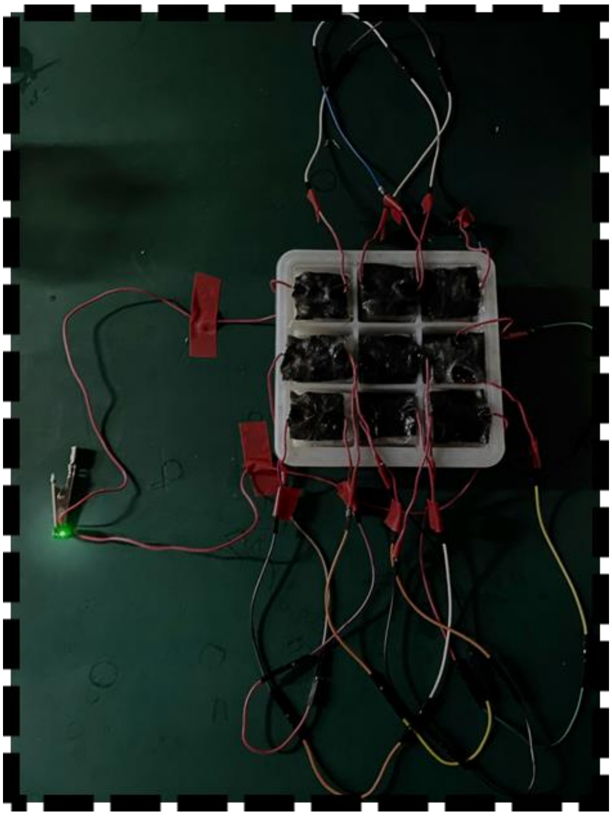

Figure S28. Optical picture of the nine-grid integrated evaporation/generator lighting

LED in dark.

References:

[1] F. Yu, Z. Chen, Z. Guo, M. S. Irshad, L. Yu, J. Qian, T. Mei, X. Wang, *ACS Sustainable Chemistry & Engineering* **2020**, *8*, 7139.

[2] Y. Yang, W. Que, J. Zhao, Y. Han, M. Ju, X. Yin, *Chemical Engineering Journal* **2019**, *373*, 955.

[3] J. Zhou, Y. Gu, P. Liu, P. Wang, L. Miao, J. Liu, A. Wei, X. Mu, J. Li, J. Zhu, *Advanced Functional Materials* **2019**, *29*, 1903255.

[4] B. Hou, Z. Shi, D. Kong, Z. Chen, K. Yang, X. Ming, X. Wang, *Materials Today Energy* **2020**, *15*, 100371.

[5] Z. Guo, J. Wang, Y. Wang, J. Wang, J. Li, T. Mei, J. Qian, X. Wang, *Chemical Engineering Journal* **2022**, *427*, 131008.

[6] Q. Zhang, X. Xiao, G. Zhao, H. Yang, H. Cheng, L. Qu, W. Xu, X. Wang, *Journal of Materials Chemistry A* **2021**, *9*, 10945.
